# Changes in major catechins, caffeine, and antioxidant activity during CTC processing of black tea from North East India†

**DOI:** 10.1039/d0ra09529j

**Published:** 2021-03-19

**Authors:** Himangshu Deka, Podma Pollov Sarmah, Arundhuti Devi, Pradip Tamuly, Tanmoy Karak

**Affiliations:** Biochemistry Department, Tocklai Tea Research Institute Jorhat 785008 Assam India himangshu1234@gmail.com; Resource Management and Environment Section, Institute of Advanced Study in Science and Technology Guwahati 781035 Assam India; Upper Assam Advisory Centre, Tea Research Association Dikom 786101 Assam India

## Abstract

Tea (*Camellia sinensis* L.) leaves undergo complex chemical transformations during black tea processing. However, the dynamic chemical changes during tea processing have not been explored in popular cultivars of North East India. In this study, changes in catechins, caffeine, total polyphenol (TP) and formation of theaflavins were examined throughout the different stages of CTC (curl, tear and crush) black tea processing based on UPLC metabolomic analysis along with antioxidant activity for eight cultivars *viz.* S.3A/3, TV1, TV7, TV9, TV17, TV22, TV23 and TV25. The results demonstrated that the most prolific changes were observed after complete maceration of tea leaves. The total catechin, (−)-epigallocatechin gallate and (−)-epicatechin gallate levels decreased by 96, 97 and 89%, respectively as the processing progressed from fresh leaves to black tea. The TP level decreased by 26 to 37% throughout the processing path. The caffeine content increased by 18% during processing. The total theaflavin reached the highest level at 20 min of fermentation and then decreased by 13 to 36% at 40 min. Cultivar TV23 and S.3A/3 had a high content of total theaflavin with 17.9 and 16.9 mg g^−1^, respectively. The antioxidant activity was observed to be decreased by 31% for the black tea as compared to fresh leaves. It is also observed that the total phenolic content exerted a greater effect on antioxidant activity rather than catechins and theaflavins. This study provides an insightful observation of black tea processing which will immensely help in improving the quality of processed tea.

## Abstract 2

Changes in catechins, caffeine, total polyphenol, theaflavins, and antioxidant activity during CTC processing of black tea from North East India cultivar were studied. Total polyphenol decreased up to 37% with the formation of theaflavins up to 1.8%.

## Graphical abstract

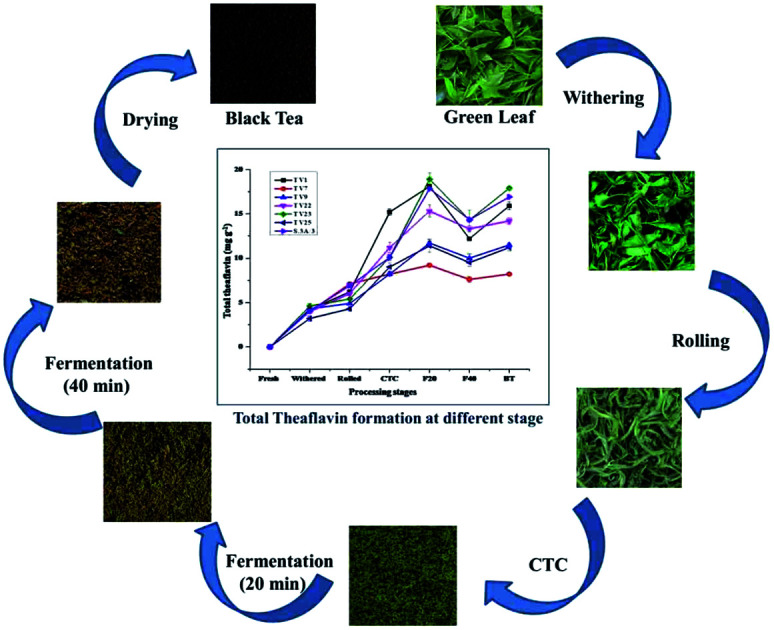

## Introduction

**Fig. 1:**
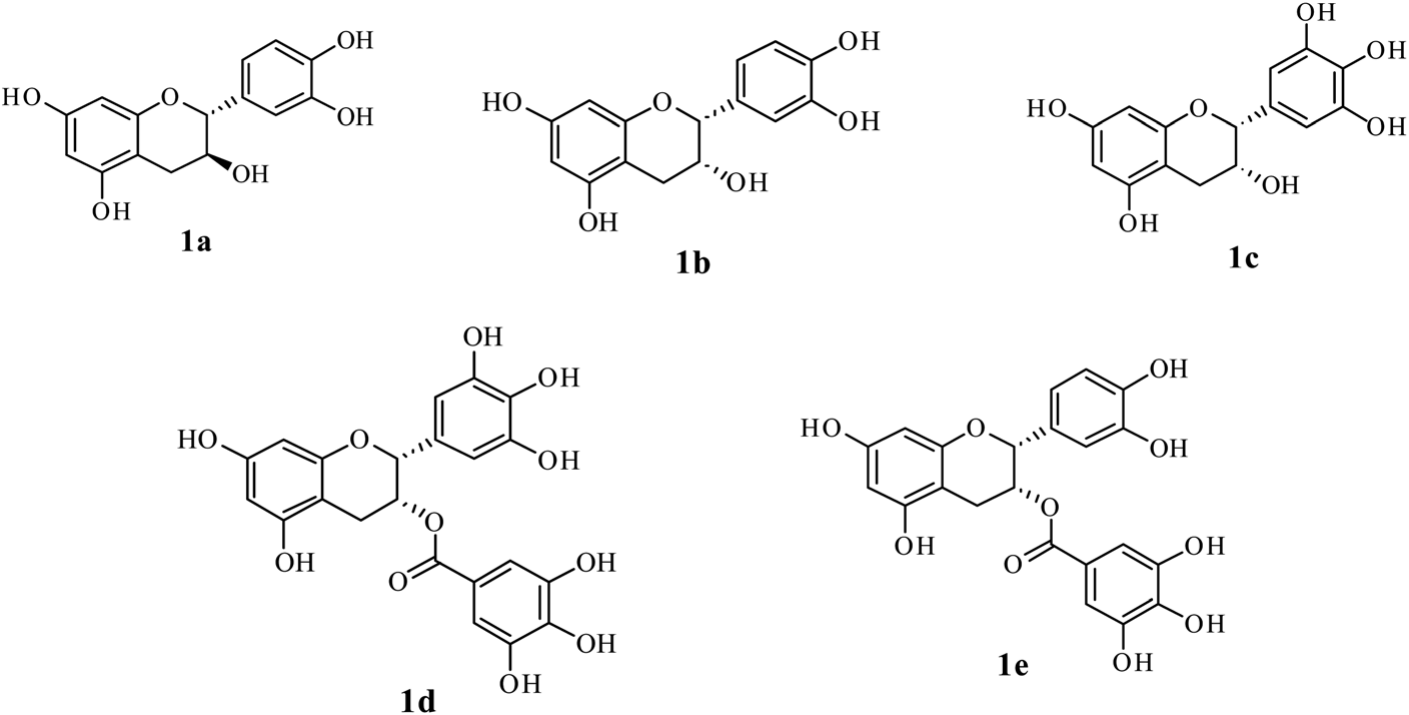
Chemical structure of major catechins in tea. (1a) (+)-catechin; (1b) (−)-epicatechin (EC); (1c) (−)-epigallocatechin (EGC); (1d) (−)-epigallocatechin gallate (EGCG); and (1e) (−)-epicatechin gallate (ECG).

The unique aroma, taste and health benefits of tea, processed from the young shoots (comprising the apical bud and 2 or 3 young leaves) of *Camellia sinensis* (L.), have promoted this drink worldwide.^1^ The properties like aroma, taste, color and health benefits are the main determining factors for the quality of processed tea.^2^ Non-volatile components are primarily responsible for color, taste and health benefits.^3^ Factors such as cultivars, climate, seasons, shading, soil conditions, elevation, *etc.* affect the content of non-volatile compounds in young shoots used for tea processing.^4,5^ Compounds contributing to the quality of tea infusion vary between harvest period and cultivar.^4,6^ Catechins, the major quality phenolic compounds in tea, represent 10 to 25% of fresh leaf dry weight.^7,8^ The principal catechins contributing 50 to 80% to total catechin (TC) are (+)-catechin, (−)-epigallocatechin (EGC), (−)-epicatechin (EC), (−)-epigallocatechin gallate (EGCG) and (−)-epicatechin gallate (ECG)^9,10^ (Fig. 1).

Tea leaves can be processed through different manufacturing protocols and six major types of tea *viz.* green, black (CTC: curl, tear and crush and orthodox), oolong, dark, yellow and white teas can be prepared. Each one has distinct metabolic profiles.^11^ Among these processed teas, black tea only accounts for more than 55% of total worldwide tea production^12^ which is the most consumed tea worldwide.^13^ India, the second-largest producer, contributed more than 21% to worldwide tea production in 2018 and the third-largest exporter of tea after Kenya and Sri Lanka.^12,14^ Assam, a state of North East (NE) India, is the major tea-producing region of India covering an area of 0.32 million ha and contributes more than 51% to the total Indian tea production.^15,16^ The tea produced in Assam is widely consumed as India accounts for 19% of global consumption becoming the second-largest consumer.^12^ According to Food and Agriculture Organization of the United Nations, the Assam tea is also exported worldwide which is evident in India's tea export data.^12^

**Scheme 1:**
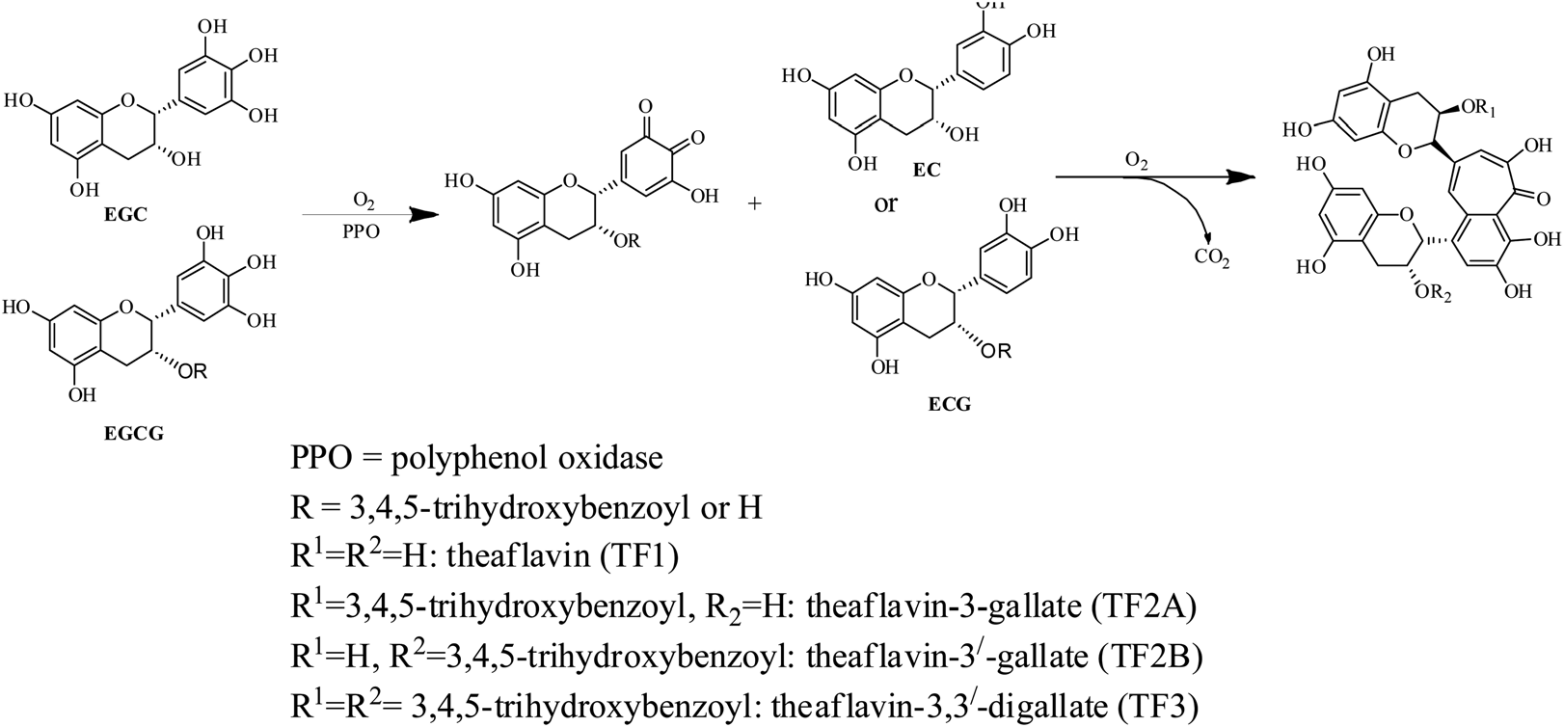
Oxidation and condensation of catechins in tea to form major theaflavins

The complex chemical composition of tea infusion necessitates the understanding of chemical changes during each stage of processing for improvement of tea quality.^17^ CTC black tea processing involves five principal stages comprising withering, rolling, CTC, fermentation and drying.^18–20^ The quality potential of black tea is also dependent on the plucking of leaves when aroma precursors are developed.^2^ Furthermore considerable chemical changes take place at each processing stage. The catechins, also called flavanols, are oxidised into more complex polymeric compounds like theaflavins (Scheme 1), thearubigins, theacitrins, theasinensins, theanaphthoquinones, *etc.*^21,22^ Theaflavins are a group of polyphenolic compounds containing more than 20 theaflavin derivatives.^23^ The principal theaflavins reported in black tea are theaflavin (TF1), theaflavin-3-gallate (TF2A), theaflavin-3′-gallate (TF2B), and theaflavin-3,3′-digallate (TF3). Information on individual theaflavin rather than the total content has been more useful in assessing tea quality. Theaflavins are critical to the quality of black tea as they contribute to color, brightness, sensory characteristics and cream formation of liquor.^24^ Theaflavins also contribute to the astringency of the liquor which varies with changes in galloylation of theaflavin. The relative abundance of these fractions varies from tea to tea.^25^ Theaflavins also have an immense contribution to the bioactivity of tea. The numerous health beneficial effects of theaflavins include antibacterial, antidiabetic, anti hemolytic, antihepatitis C virus infection, antiinflammatory and anticancer effects.^26–28^ Even, in some cases effect of theaflavins exceeds the catechins. For example, the antibacterial activity of TF3 is stronger than that of EGCG.^26^

Tocklai Tea Research Institute (TTRI), India has developed 35 vegetative propagated tea cultivars and these cultivars are being extensively used by the tea industry in NE India as planting material for black tea production.^4^ The biochemical characterization of young fresh leaves of these cultivars coupled with their variation with the season had been reported in earlier studies.^4,29^ However, reports on black tea processing from the young leaves of these cultivars are very scanty. Therefore, to enlighten the black tea processing from the biochemical aspect, this investigation aims to determine the changes in catechin, caffeine, and total polyphenol (TP) content with the formation of theaflavins, and the variation of antioxidant activity at different stages of black tea processing.

## Materials and methods

### Chemicals

Gallic acid monohydrate (≥98.0%), (−)-epigallocatechin-3-gallate (≥95%),(−)-epigallocatechin (≥95%, HPLC), (−)-epicatechin-3-gallate (≥95%, HPLC), (+)-catechin (≥95%, HPLC), (−)-epicatechin (≥90%, HPLC), caffeine (anhydrous, 99%), theaflavin (≥90%, HPLC), theaflavin-3-gallate (≥95%, HPLC), theaflavin-3′-gallate (≥90%, HPLC), theaflavin-3,3′-digallate (≥90%, HPLC), 2,2-diphenyl-1-picrylhydrazyl (DPPH), 6-hydroxy-2,5,7,8-tetramethylchroman-2-carboxylic acid (Trolox), 2,2′-azinobis(3-ethylbenzothiazoline-6-sulfonic acid) diammonium salt (ABTS), potassium persulfate, 2,4,6-tri(2-pyridyl)-*s*-triazine (TPTZ) and iron(iii) chloride hexa-hydrate were purchased from Sigma-Aldrich, India. Folin-Ciocalteu reagent (LR) was obtained from Himedia (HiMedia Laboratories, India). Acetonitrile (HPLC grade), acetic acid (HPLC grade), sodium carbonate and all other chemicals were procured from Merck KGaA, Darmstadt, Germany.

### Instrumentation

A Dionex, Ultimate 3000 UPLC system was used for the determination of catechin, caffeine and theaflavin. The UPLC system was fitted with Luna 5 μ phenylhexyl phenomenax column (4.5 mm × 250 mm) which is maintained at 25 ± 0.5 °C and a UV-Vis detector. For catechin and caffeine, the detector wavelength was set at 278 nm, whereas it was 380 nm for theaflavin. A Varian, Cary 50 Conc spectrophotometer was used for UV-Vis spectroscopy. For water used in the experiments, a Millipore Milli-Q Synthesis water purifier from Merck, Germany was used. For rolling of tea leaves, Pizey rolling table (1 kg capacity) was used. CTC of rolled leaves was done in a CTC roller with 10 TPI (teeth per inch). For fermentation, a Galaxy 170 R (Model No. CO170R-230-0200) from New Brunswick (an Eppendorf company) was used. A fluid bed drier (FBD) from Teacraft, United Kingdom was used for drying the fermented samples.

### Tea leaf sampling

Considering the major genetic variations of NE Indian tea cultivars and their popularity in tea industry^4,30^ eight cultivars were chosen for the study. The cultivars were S.3A/3 (Assam variety); TV1 and TV17 (Assam-China hybrid variety); TV7 (China hybrid variety); and TV9, TV22, TV23, and TV25 (Cambod variety). Young tea shoots containing the apical bud and first two leaves of the above-mentioned cultivars grown under same cultivation practices, soil nutrient management and bush age in the range from 30 to 35 years, were hand-plucked from Borbheta experimental Tea Estate (T.E.) of TTRI, Assam, India during July and August of 2017. This estate is located between 26°43′14′′N and 94°11′54′′E with an elevation of 96.5 meters above the mean sea level. The meteorological data of the estate during 2017 and the origin of the cultivars can be obtained from our recent work.^4^

### Black tea processing and sample preparation

The CTC black tea was processed using the environment-controlled manufacturing (ECM) unit obtained from Teacraft, United Kingdom. For the first step of CTC black tea processing *i.e.* withering, 1.5 kg fresh leaves from each cultivar were evenly spread in a trough with a thickness of around 2 cm and kept for around 16 to 18 h at room temperature. When the moisture level of the leaves reached 68 to 70% from the initial 76 to 78%, the leaves were subjected to maceration. The maceration technique involved rolling for 15 min followed by 3 cut CTC (CTC operation for three times consecutively). The macerated leaves were allowed to undergo fermentation for 60 min. The fermentation chamber was kept at temperature 30 ± 2 °C and 95% relative humidity. The fermented tea samples are then dried at two steps, starting at 120 ± 2 °C for 10 min followed by 90 ± 2 °C for 10 min. For experimental analysis, 10 g of sample was collected at each stage of fresh leaf, withering, rolling, CTC, 20 and 40 min of fermentation (F20 and F40), and finally produced black tea (BT). The stepwise collected samples, except BT, were steamed for 1 min, immediately after the sampling, for deactivation of polyphenol oxidase (PPO) and peroxidase. These steamed samples were dried at 60 ± 2 °C for 1 h and kept at −20 °C for further analysis.

### Determination of total polyphenol contents

TP contents were determined according to the ISO 14502-1:2005 method described by the International Standard Organization.^31^ Briefly, 0.20 g of ground tea leaf sample was extracted with 5 mL 70% methanol at 70 °C in a water bath for 10 min and allowed to settle down. The supernatant extract was transferred to a 10 mL volumetric flask. The process was repeated one more time. The final volume was made up to 10 mL by adding 70% methanol. 1 mL of this extract was diluted to 100 mL with water in a volumetric flask. 1 mL of the diluted extract was transferred into a test tube and 5 mL 10% (v/v) Folin–Ciocalteu reagent was added to it with vigorous stirring. After 3 min, 4 mL of 7.5% (w/v) sodium carbonate solution was added to the reaction mixture and mixed using a vortex. The reaction mixture was allowed to stand at room temperature for 60 min. The absorbance of the resultant mixture was measured at 765 nm in the UV-Vis spectrophotometer. A sample blank using water instead of tea extract was also measured. The measurement was done against a gallic acid calibration curve (*y* = 0.012*x* − 0.046, *R*^2^ = 0.999) prepared using concentrations in the range from 10 to 50 μg mL^−1^.

### Determination of catechin and caffeine contents

Catechin and caffeine contents were determined using ISO 14502-2:2005 method of the International Standard Organization method.^32^ The extraction was done as described in the determination of TP to a 10 mL volumetric flask. 1 mL of the resultant extract was diluted with the stabilizing agent to a 5 mL volumetric flask. The stabilizing agent was prepared by using ascorbic acid (500 μg mL^−1^), EDTA (500 μg mL^−1^), and acetonitrile (25% v/v) in water. The diluted extract was filtered through 0.45 μm syringe filters before quantitative determination using UPLC. The column was eluted using two solvent systems consisting of 2% (v/v) acetic acid, 9% (v/v) acetonitrile in water (A) and 80% (v/v) acetonitrile in water (B). Gradient elution was set as 100% mobile phase consisted of A for 10 min followed by a linear gradient to 68% of A and 32% of B over 15 min and held at this composition for 10 min. The flow rate was 1 mL min^−1^. The peaks were identified by comparing with catechins and caffeine standard peak. The quantitative determination of individual catechins and caffeine were done by using relative response factors of catechins with respect to caffeine as described in ISO 14502-2:2005.^32^

### Determination of total theaflavin by spectrophotometric methods

Total theaflavin of tea infusions was estimated using the method developed by Ullah.^33^ Briefly, 6 g tea was taken in a thermo-flask and 250 mL boiling water was poured with intermittent shaking. After 10 min, tea samples were filtered off and the filtrate (called infusion) was allowed to cool down. 6 mL of the infusion was mixed with an equal volume of 1% (w/v) Na_2_HPO_4_. To this mixture 10 mL ethyl acetate was added and shaken vigorously for 1 min and again 5 mL ethyl acetate was added to it. Then 10 mL ethyl acetate extract was poured into a 25 mL volumetric flask and the volume was made up with methanol. The absorbance of this extract was read at 380 nm against a reference solution of ethyl acetate and methanol in a 1 : 1.5 ratio. The total theaflavin content was calculated using the following formulaTotal theaflavin (%) = 2.25 × absorbance

### Determination of theaflavin using UPLC

**Fig. 2:**
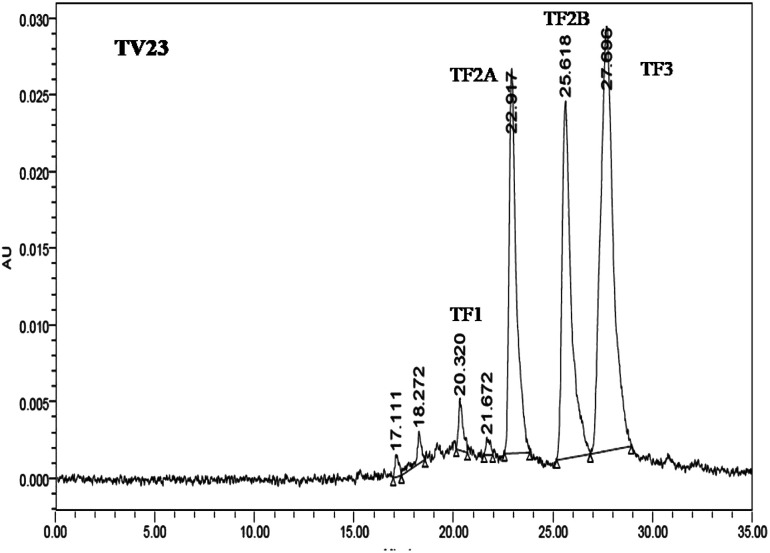
A typical UPLC chromatograph of theaflavins extracted from TV23 black tea.

The quantification of individual theaflavins was carried out using the method described in Opie *et al.*^34^ Theaflavins were extracted from tea infusion prepared by brewing 6 g tea in 250 mL boiling water for 10 min. To 50 mL of the cold infusion, 50 mL ethyl acetate was added and shaken slowly for 10 min. The ethyl acetate extract was washed with 2% NaHCO_3_ for the removal of low molecular weight thearubigins. 20 mL ethyl acetate extract was dried using a rotary evaporator at a temperature around 50 °C. The dried extract was quantitatively dissolved in 10 mL methanol. The samples were filtered through a 0.45 μm cellulose acetate filter before UPLC analysis. The filtered extract (20 μL) was injected into the UPLC column. The solvent system consisted of two solvents: solvent A (0.5% acetic acid) and solvent B (0.5% acetic acid and 30% acetonitrile). The elution was set as 100% solvent A followed by a linear gradient to 100% solvent B for 45 min with a flow rate of 1 mL min^−1^. The identification and determination of the content of individual theaflavins were done using individual standards. A typical chromatograph of theaflavins extracted from TV23 black tea is presented in Fig. 2.

### Preparation of extracts for antioxidant activity

The major components in tea can be extracted with water in boiling conditions. 5 g finely ground tea sample was boiled in distilled water for 3 min on a hot plate and then filtered. The filtrate volume was adjusted to 50 mL. This tea infusion was centrifuged at 10 000 rpm for 10 min to remove suspended matters. The supernatant was used for further analysis.

### DPPH radical scavenging activity

Free radical scavenging activity assessment, using DPPH, was performed by the method of Brand-Williams *et al.*^35^ with a slight modification. Briefly, 2.5 mL of 1 mM ethanolic solution of DPPH was mixed with 2.75 mL tea extract ethanolic solution (11 fold diluted). The mixture was shaken vigorously and kept at room temperature for 30 min in dark condition. The absorbance was read at 517 nm using a UV-Vis spectrophotometer. This activity is termed as percent DPPH scavenging activity and calculated asDPPH inhibition (%) = [(*A*_B_ − *A*_E_)/*A*_B_] × 100where *A*_B_ is the absorbance of the blank sample, and *A*_E_ is the absorbance of the reaction mixture.

A standard curve with various concentrations of Trolox was also prepared and the results were expressed in mmol Trolox equivalent per gram of tea.

### Ferric reducing antioxidant potential (FRAP) assay

The ferric reducing ability of tea extracts was determined using the method of Benzie and Strain^36^ with slight modification. The reduction was measured by the absorption difference at 593 nm. The working reagent was prepared freshly by mixing 300 mM acetate buffer (pH 3.6), 10 mM TPTZ in 40 mM hydrochloric acid and 20 mM ferric chloride in the ratio of 10 : 1 : 1 (by volume). Different concentrations of FeSO_4_·7H_2_O were used to prepare the standard curve. The tea extracts were diluted 1000 times with water before analysis. 7 mL of the working reagent was added to 0.5 mL of diluted tea extract, followed by incubation in a water bath at 37 °C for 30 min. A sample blank was also prepared using acetate buffer. The difference in absorbance at 593 nm was used to calculate the FRAP activity. The FRAP activities were expressed as mol Fe^2+^ per gram of tea.

### Statistical analysis

Analysis of variance (ANOVA) followed by Tukey's multiple comparison test was used to get the differences between means and the differences were considered significant at *p* ≤ 0.05 and *p* ≤ 0.01. For each sample, all data were reported as the mean ± standard error (SE) with three replications. Pearson correlations were drawn among the different parameters to study the relationship among them. All the statistical analyses were carried out using SPSS software version 17.00 (SPSS Inc., Chicago, IL).

## Results and discussion

**Fig. 3:**
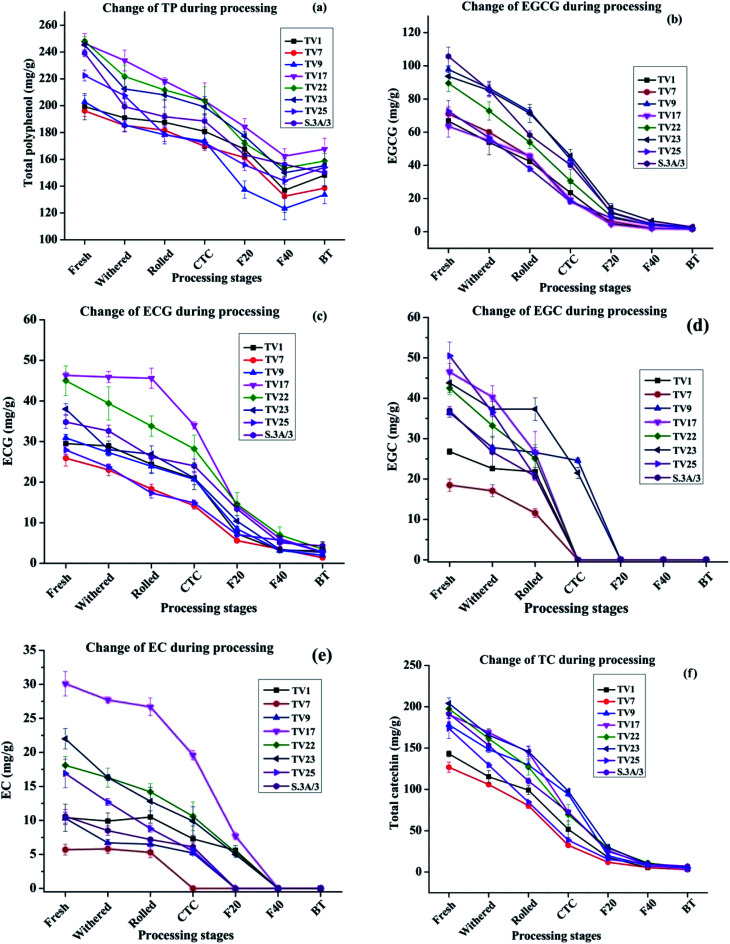
Changes in catechin levels from fresh leaves to black tea during processing: (a) total polyphenol (b) epigallocatechin gallate (EGCG), (c) epicatechin gallate (ECG), (d) epigallocatechin (EGC), (e) epicatechin (EC) and (f) total catechin (TC). Fresh, fresh leaves; withered, withered leaves; rolled, rolled leaves; CTC; CTC leaves; F20, leaves at 20 min of fermentation; F40, leaves at 40 min of fermentation; and BT, black tea.

The changes in TP level during black tea processing for different cultivars are presented in Fig. 3(a). The level of TP was found to decrease in the range from 25.7% in TV1 to 37.3% in S.3A/3 in the course of processing from fresh leaves to BT. This variation in decrease led to a difference in TP level in black teas processed from leaves having similar TP levels. This difference in TP level in BT might be originated from different compositions of catechins in fresh leaves although having a similar level of TP. The TP content in BT samples produced varied from 133.6 to 167.6 mg g^−1^ with the highest content in TV17. Except for TV1, the TP content in BT is significantly different (*p* ≤ 0.05) from fresh, withered, rolled, and CTC stages. The decrease was very rapid during the fermentation stage indicating fast oxidation of catechins. With the progress of processing, biochemical compositions undergo substantial changes. During withering a large number of physicochemical changes such as a decrease in moisture level, increased permeability of cell membrane, the formation of volatile flavor compounds, *etc.* occur. The rolling of green leaf crushes the cell ultrastructure of withered leaves exposing the polyphenols, which leads to fast enzymatic oxidation of these compounds. During the fermentation, PPO oxidizes the polyphenols rapidly to more complex polyphenols such as theaflavin, thearubigins, *etc.* The chemical characteristics of these polyphenols formed during the oxidation processes are different from fresh leaves.^37^

The NE India tea, especially the Assam tea, is known for its body, briskness and bright colour contributed mainly by theaflavin. The tropical climate of the region contributes to the unique malty taste of tea produced here. The behavior of chemical changes during CTC processing of black tea from NE Indian tea cultivar is similar to cultivars from other regions of the world.^37,38^ For instance, similar to the present study, Lee *et al.*^37^ observed a near complete degradation of major catechins during the processing of black tea. However, the extent of changes in TP contents was different. In the present study, the decrease in TP content (25–37%) was much less than that processed in Korea (53%).^37^ Most studies in this line showed the greatest change of catechin and theaflavin content during the fermentation stage.

The extent of oxidative degradation varied for each catechin. EGCG and ECG levels at different stages of black tea processing are presented in Fig. 3(b) and (c), respectively. The EGCG and ECG levels for all the cultivars decreased by >97% and >89%, respectively on going from fresh leaves to BT during the manufacturing process. In conformity with earlier available literature,^38–40^ the observed decrease in the content of phenolic compounds in this study was very rapid during the fermentation stage. EGCG decreased more than that of ECG during fermentation. Owuor and Obanda observed that trihydroxy catechins (EGCG and EGC) were oxidized faster than their dihydroxy counterparts (ECG and EC).^41^ The availability of three hydroxyl groups on the B ring of trihydroxy catechins makes it more convenient to lose a hydrogen atom forming a semiquinone radical with an oxygen atom possessing an unpaired electron.^39^ Moreover, the semiquinone anion radical of trihydroxy catechins is more stable than that of dihydroxy catechins due to the presence of an additional oxygen atom that can stabilize the negative charge on the adjacent carbon by inductive effect (resonance structures are shown in Scheme 1, ESI†). The EGCG level was found to decrease in the range from 77.5% (TV25) to 91.1% (TV1) whereas the ECG level decreased in the range from 61.7% (TV25) to 84.7% (TV1) with the change of processing stage from CTC to F40. EGCG content in fresh leaves, rolled, CTC and F20 are significantly different (*p* ≤ 0.05) from each other. However, no significant difference was observed after the F20 stage as 68% (on average) of the initial content was consumed in oxidative degradation. From rolling to F40, the ECG level at each stage is significantly different (*p* ≤ 0.05) from that in the previous stage. The cleavage of the galloyl groups, before theaflavin formation, may be one of the factors for the decreasing content.^39^ The EGC and EC levels at different processing stages are presented in Fig. 3(d) and (e), respectively. The EGC and EC levels could not be detected in black tea indicating their complete oxidation or conversion. The TC levels for all the cultivars decreased by >96% on going from fresh leaves to BT [Fig. 3(f)]. On going from CTC to F40, TC level decreased in the range from 74.9% (TV25) to 91.9% (TV9). Specifically, from rolling to F40 stage, TC level is significantly different (*p* ≤ 0.05) at every stage compared to the previous stage. However, no significant difference was observed in TC content in BT and F40 stage.

**Fig. 4:**
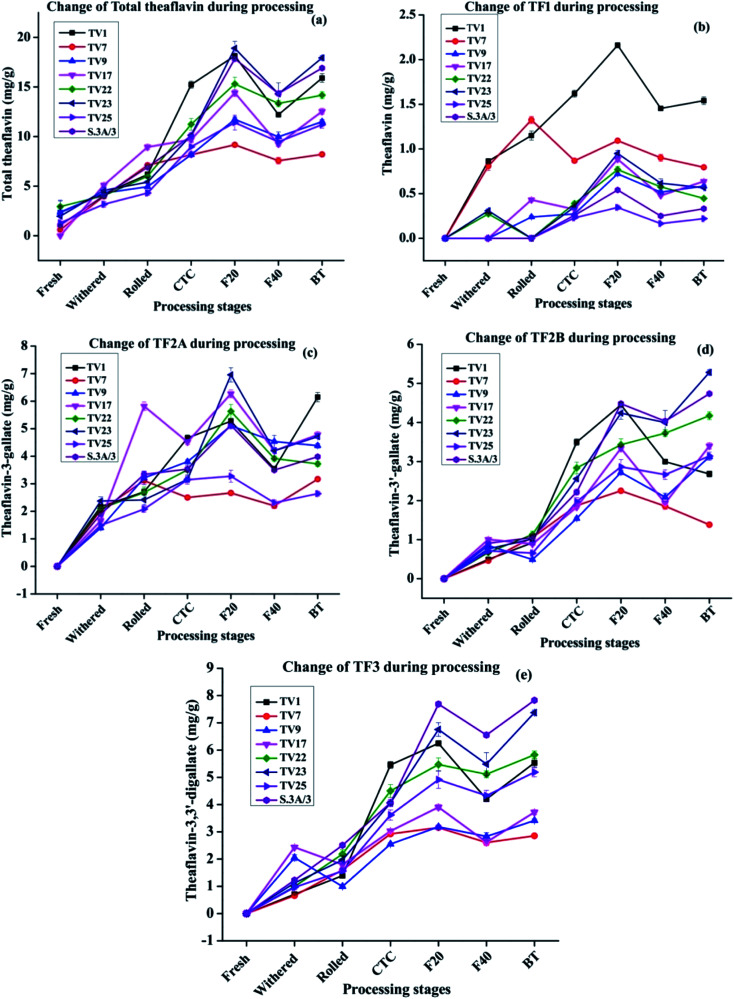
Formation of principal theaflavins during black tea processing: (a) total theaflavin, (b) theaflavin (TF1) (c) theaflavin-3-gallate (TF2A) (d) theaflavin-3′-gallate (TF2B) (e) theaflavin-3,3′-digallate (TF3). Fresh, fresh leaves; withered, withered leaves; rolled, rolled leaves; CTC; CTC leaves; F20, leaves at 20 min of fermentation; F40, leaves at 40 min of fermentation; and BT, black tea.

During black tea processing catechins in fresh leaves are oxidized and polymerized to form theaflavins and thearubigins, which are critically important towards the quality of black tea.^42,43^ PPO acts as the catalyst for the *in vitro* oxidative conversion of catechins into theaflavins.^44^ In tea leaves, PPO is accumulated in chloroplast whereas the vacuole stores the phenolic compounds.^23^ The maceration of tea leaves accelerates the PPO catalyzed catechin oxidation by oxygen leading to the formation of quinones. These quinones, resulting from the oxidation of B-ring of both dihydroxy and trihydroxy catechins, condense to form different theaflavins^45^ which imparts orange color to the infusion of black tea^13,23^ The content of total theaflavin at different processing stages is presented in Fig. 4(a). The formation of individual theaflavins was observed from the withering stage with an increasing trend. Theaflavins get synthesized very rapidly once the rolled leaves are subjected to CTC. Microstructural changes of the cell walls or cellular membranes during CTC expose the cellular components to PPO which led to the accelerated formation of theaflavins. The total content of theaflavin reached the highest value at the F20 and then decreased by 12.9 to 35.9% at the F40. This decrease in total theaflavin content was significant (*p* ≤ 0.05) for cultivars TV1, TV9, TV17, TV23, and S.3A/3. This may be due to further oxidation of some amount of theaflavins formed during the initial period of fermentation by epicatechin quinone to form thearubigins.^13,37,45,46^ Besides, with the increase in fermentation duration, theaflavin is oxidized to theanaphthoquinone.^47^ Finally, the total theaflavin content again increased by 6.3 to 35.7% in black tea. This increase was significant (*p* ≤ 0.05) for cultivars TV1, TV9, TV17, TV23, and S.3A/3. Tea processed from the cultivar TV23 and S.3A/3 had a higher content of total theaflavin with 17.9 and 16.9 mg g^−1^, respectively. These high theaflavin contents can be justified by the higher contents of catechins in the fresh leaves of the cultivars. The TC and individual catechins had a significant (*p* ≤ 0.01) negative correlation with total theaflavin for all cultivars with progress of the processing (ESI, Tables S1A–H†). TF1, TF2A, TF2B, and TF3 levels at different stages of black tea processing are presented in Fig. 4(b)–(e), respectively. Black tea processed from cultivar TV1 had the highest content of TF1 with 1.54 mg g^−1^ and that of cultivar TV25 had the lowest content with 0.22 mg g^−1^. TF1 content at F20 is significantly different (*p* ≤ 0.01) from the other stages for all cultivars. TF2A content was found to be the highest in black tea processed from cultivar TV1 (6.1 mg g^−1^). Cultivar TV17 and TV23 had equal content (4.7 mg g^−1^) of TF2A. TF2A content in F20 for cultivars TV1, TV9, TV22, TV23, and S.3A/3 was significantly different (*p* ≤ 0.05) from the other processing stages. TF2B formation was observed highest in black tea processed from cultivar TV23 (5.28 mg g^−1^) and S.3A/3 (4.74 mg g^−1^). The TF2B levels in F20 of black tea processing for cultivars TV1, TV7, TV9 and S.3A/3 were significantly different (*p* ≤ 0.05) from the other stages. These cultivars along with TV23 had significantly different (*p* ≤ 0.05) level of TF2B in black tea. TF3 level was highest in S.3A/3 (7.83 mg g^−1^) black tea followed by TV23 (7.37 mg g^−1^). Except TV1 cultivar, the TF3 level in black tea significantly differ (*p* < 0.05) from other processing stages excluding F20. The higher level of TF3 had a significant negative correlation (*p* ≤ 0.01) with total catechin as well as individual catechins at each stage of processing (ESI, Tables S1A–H†). Total theaflavin as well as individual theaflavin contents were observed lowest in black tea processed from cultivar TV7 which is a China variety with low phenolic contents.

The cultivars showed the same trend in terms of polyphenols variation, with monomeric catechins decreasing and polymeric catechins increasing over the various processing steps. However, some cultivars exhibited anomalous behavior in specific steps, for instance, the theaflavin content in TV1 increases drastically during the first 20 min of fermentation. The difference in behavior in theaflavin formation originated from specific properties of the cultivar. The cultivar TV1 has higher PPO activity as compared to other cultivars.^48^ This higher PPO activity led to a comparatively higher amount of theaflavin formation in the early part of fermentation.

The composition and distribution of individual catechins are more important than its TC for the formation of theaflavins which influences the quality of processed black tea.^41,44^ The high content of trihydroxy catechins and low content of dihydroxy catechins is critical to the quality of black tea.^41^ The maximum theaflavin formation is ensured by the correct balance and content of trihydroxy and dihydroxy catechins. The trihydroxy catechins are oxidized faster during fermentation owing to their lower redox potentials. Therefore, trihydroxy catechins can be the limiting factor for theaflavin formation as they are ended up faster.^41^ In the present study, cultivars TV22, TV23 and S.3A/3 with a higher content of galloylated catechins (EGCG and ECG) resulted in the synthesis of a higher amount of TF3 (5.83–7.83 mg g^−1^) which is considered as one of the quality criterion for black tea.^41^ In conformity with our results, Teng *et al.* in a model study of theaflavin synthesis using PPO enzymatic reaction demonstrated that a high concentration of EGCG and ECG led to a higher level of TF3 synthesis.^44^ This higher level could be justified by the fact that the galloylated catechins upon hydrolysis release gallic acid, which is then get inserted into theaflavins to form TF3.

The relative abundance of the theaflavin monogallate and digallate affect the taste in terms of briskness. This study provides the theaflavin profile of eight popular cultivars of NE India tea industry. Based on this information black tea with a specific theaflavin profile can be processed. Moreover, the level of gallated theaflavins can be enhanced by regulating the temperature, air (O_2_), withered leaf moisture along with the maceration technique.

**Fig. 5:**
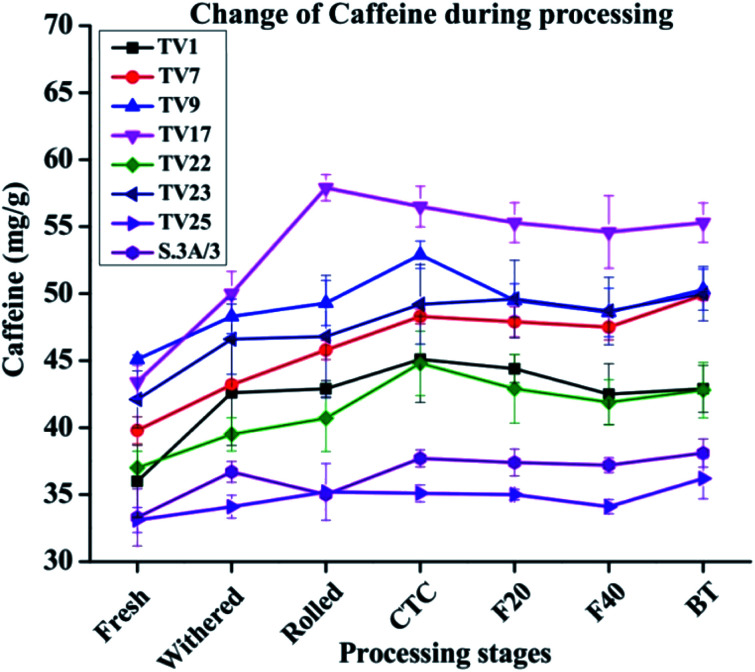
Changes in caffeine levels from fresh leaves to black tea during processing. Fresh, fresh leaves; withered, withered leaves; rolled, rolled leaves; CTC; CTC leaves; F20, leaves at 20 min of fermentation; F40, leaves at 40 min of fermentation; and BT, black tea.

The caffeine content increased between 1.09 and 1.27 fold during the processing of black tea compared to fresh leave with the highest change in TV17 and the lowest in TV25 (Fig. 5). The increase was prominent during the withering stage. The tea shoots during withering continue to respire at the expense of the sugar reserve of the shoots and caffeine is being synthesized during the withering period which results in increased caffeine content.^49^ The increase in caffeine content might have a relation to amino acid metabolism.^50^ The breakdown of nucleic acids results in the formation of caffeine which continues during the withering of tea shoots leading to an increase in caffeine content in black tea.^51^ An increase in caffeine level during black tea processing has been reported by earlier studies also.^40,51,52^ The caffeine level at the withered stage was observed to increase by 3% (TV25) to 18% (TV1). No significant change was observed during fermentation for all the cultivars. In accordance with the present observation, Sari and Velioglu^51^ observed a significant increase in caffeine level during the withering stage of Turkish black tea. However, unlike Turkish tea, the caffeine level in Indian tea was also observed to be increased in steps other than withering, especially rolling and CTC. Previous studies also reported a very minimal effect of fermentation on caffeine levels during processing.^40,53^ In the present study, the enhanced level during withering led to a higher content of caffeine than that in fresh leaves.

**Fig. 6:**
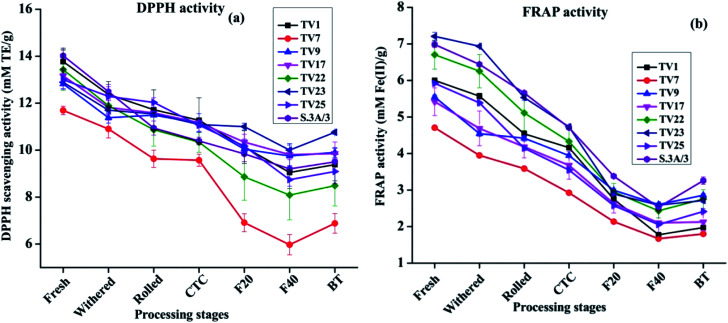
Changes in antioxidant activity from fresh leaves to black tea during processing: (a) DPPH scavenging activity, and (b) FRAP activity. Fresh, fresh leaves; withered, withered leaves; rolled, rolled leaves; CTC; CTC leaves; F20, leaves at 20 min of fermentation; F40, leaves at 40 min of fermentation; and BT, black tea.

Theaflavins have been demonstrated to have strong antioxidant activities both *in vivo* and *in vitro*, in several reports.^54,55^ These physiological properties have drawn widespread attention towards theaflavins.^44^ Antioxidant activity was assessed at each stage of black tea processing using free radical scavenging activity (DPPH and ABTS as model free radical) and ferric reducing antioxidant potential (FRAP) assay. DPPH scavenging activities at different stages of processing are presented in Fig. 6(a). The activity reduced by 17 to 41% on going from fresh leave to BT. The highest activity in fresh leaves can be attributed to the highest phenolic contents in fresh leaves compared to other stages. The antioxidant activity exhibited a significant positive correlation with phenolic contents (ESI, Tables S2A–H†). The activity in each stage of manufacturing followed the same trend as observed in the case of TP in the present study. Fresh leaves from cultivar S.3A/3 showed the highest DPPH scavenging activity with 14.02 mM TE g^−1^, whereas black tea processed from cultivar TV23 exhibited the highest activity with 10.76 mM TE g^−1^. DPPH scavenging activity of BT samples for all cultivars was found significantly lower (*p* ≤ 0.01) than that in fresh and withered leaves. A similar trend was also observed for ABTS scavenging activity (included in the ESI†).

FRAP activity throughout the processing pathway followed the same trend as DPPH scavenging activity. Changes in FRAP activity from fresh leaves to BT are presented in Fig. 6(b). The activity in fresh leaves varied between 4.71 mM Fe^2+^ g^−1^ (TV7) and 7.21 mM Fe^2+^ g^−1^ (TV23). These activities decreased with the progress of processing by 48 to 67%. The FRAP activity in BT was in the range between 1.80 and 3.26 M Fe^2+^ g^−1^ with the highest activity in S.3A/3. The activity in BT was significantly different (*p* ≤ 0.01) from all stages before fermentation.

The results from the antioxidant assays revealed that ABTS and FRAP activity for fresh leaves was highest for cultivar TV23, whereas fresh leaves of S.3A/3 had the highest DPPH activity. In the case of black tea, cultivars TV23 and S.3A/3 had a higher level of activity in comparison to other cultivars for all three assays. The China variety cultivar TV7 showed the lowest activity. The extent of the decrease in antioxidant activity was similar for ABTS and FRAP with the progress of processing in the range from 48 to 71%, whereas the reduction in DPPH activity was comparatively less prominent (17–41%).

All the three antioxidant activity assays observed a strong positive (*p* ≤ 0.01) correlation with TC, individual catechins and TP for all cultivars (ESI, Tables S2A–H†). On the other hand, antioxidant activity showed a significant negative (*p* ≤ 0.01) correlation with total and individual theaflavins across different stages of black tea processing (ESI, Tables S3A–H†). Although theaflavin contents were observed to be increased with the progress of processing, the oxidative degradation of catechins was much faster than the formation of theaflavins leading to decreased content of TP which justifies the negative correlation of antioxidant activity with theaflavins during the processing of black tea.

## Conclusion

This study demonstrates the change in biochemical components throughout the different stages of black tea processing. The leaf maceration has been the key step regarding biochemical changes. The maceration process accelerated the oxidative degradation of catechins as well as the formation of theaflavins. The cultivars TV23, S.3A/3 and TV22 had a high content of catechins which was reflected in higher theaflavin contents in black tea processed from these cultivars. The caffeine content increased by 18% during processing. The antioxidant activity was observed highest in fresh leaves which reached a 31% decreased level at the black tea stage. The total phenolic contents had a higher effect on antioxidant activity rather than catechins and theaflavins. The insight full observation of black tea processing reported here will immensely help in processing improved quality tea, with specific criteria of theaflavin profile, in NE India.

## Author contributions

Himangshu Deka: conceptualization, methodology, investigation, formal analysis, statistical analysis, validation, writing-original draft. Podma Pollov Sarmah: investigation, validation, writing-reviewing and editing. Arundhuti Devi: writing-reviewing and editing. Pradip Tamuly: supervision, writing-reviewing and editing. Tanmoy Karak: visualization, data curation, writing-reviewing and editing.

## Abbreviations

ANOVA: Analysis of variance
BT: Black tea
CTC: Curl, tear and crush
EC: Epicatechin
ECG: Epicatechin gallate
ECM: Environment controlled manufacturing
EGC: Epigallocatechin
EGCG: Epigallocatechin gallate
F20: 20 min of fermentation
F40: 40 min of fermentation
NE: North East
PPO: Polyphenol oxidase
SE: Standard error
TC: Total catechin
TE: Tea estate
TF1: Theaflavin
TF2AT: heaflavin-3-gallate
TF2B: Theaflavin-3′-gallate
TF3: Theaflavin-3,3′-digallate
TP: Total polyphenol
TPTZ: 2,4,6-Tri(2-pyridyl)-*s*-triazine
TTRI: Tocklai Tea Research Institute
TV: Tocklai vegetative

## Conflicts of interest

The authors declare no conflict of interest.

## Supplementary Material

RA-011-D0RA09529J-s001
