# Supplementary material for: Changes in major catechins, caffeine, and antioxidant activity during CTC processing of black tea from North East India
Source: RSC Adv. 2021 Mar 19;11(19):11457–67. doi: 10.1039/d0ra09529j (PMC8695946; doi:10.1039/d0ra09529j)
Supplement: RA-011-D0RA09529J-s001 [file RA-011-D0RA09529J-s001.pdf]

Electronic Supplementary Material (ESI) for RSC Advances.
This journal is © The Royal Society of Chemistry 2021

Changes in major catechins, caffeine, and antioxidant activity during CTC processing of black tea from North East India

Himangshu Deka[a*], Podma Pollov Sarmah[a], Arundhuti Devi[b], Pradip Tamuly[a], Tanmoy Karak[c]

[a]Biochemistry Department, Tocklai Tea Research Institute, Jorhat 785008, Assam, India

[b]Resource Management and Environment Section, Institute of Advanced Study in Science and Technology, Guwahati 781035, Assam, India

[c]Upper Assam Advisory Centre, Tea Research Association, Dikom 786101, Assam, India

[*] Corresponding author E-mail addresses: himangshu1234@gmail.com

Supplementary information

1. ABTS radical scavenging activity

Free radical scavenging ability of tea extracts, using ABTS radical cation, was tested by the method of Re et al.[26] An aqueous 7 mM concentration ABTS solution was prepared. ABTS radical cation (ABTS+•) was generated by the reaction of ABTS with potassium persulfate ($K_2S_2O_8$, 2.45 mM) in dark at room temperature for 12-16 hours. Prior to use, the ABTS+• solution was diluted 50 fold with deionized water in order to obtain an absorbance from 0.3 to 0.8 at 734 nm. An appropriate solvent blank reading (A_B) was taken. 200 µL of previously 400 fold diluted tea extract was added to 6mL of ABTS+• solution and then the reaction mixture was allowed to stand in the dark for one hour. The absorbance was read at 734 nm using water as the reference. All reagents were freshly prepared. The inhibitory activity was calculated using the following equation:

$$\text{Inhibition (\%)} = [(A_B - A_E)/A_B] \times 100$$

Where A_B is the absorbance of the blank sample, and A_E is the absorbance of the reaction mixture.

A standard curve with various concentration of Trolox was also prepared and the results were expressed in molar Trolox equivalent/ g of tea.

The ABTS scavenging activity was observed to decrease from the fresh leave stage to the final black tea product. The activities throughout the processing pathway are shown in Fig. S1. For the fresh leaves from different cultivars, the activity varied in the range between 8.66 M TE/g tea leaves in TV7 and 10.85 M TE/g tea leaves in TV23. The activity levels decreased in the range from 56 to 71% at the final stage of processing in comparison to fresh leaves. For the black tea samples, this activity varied between 2.56 M TE/g (TV7) and 4.68 M TE/g (S.3A/3). The changes in ABTS scavenging activity from the fresh stage to F40 were significant ($p \leq 0.05$) for all cultivars except TV17. However, this activity of TV17 black tea sample was significantly different ($p \leq 0.01$) from the first three processing stages.

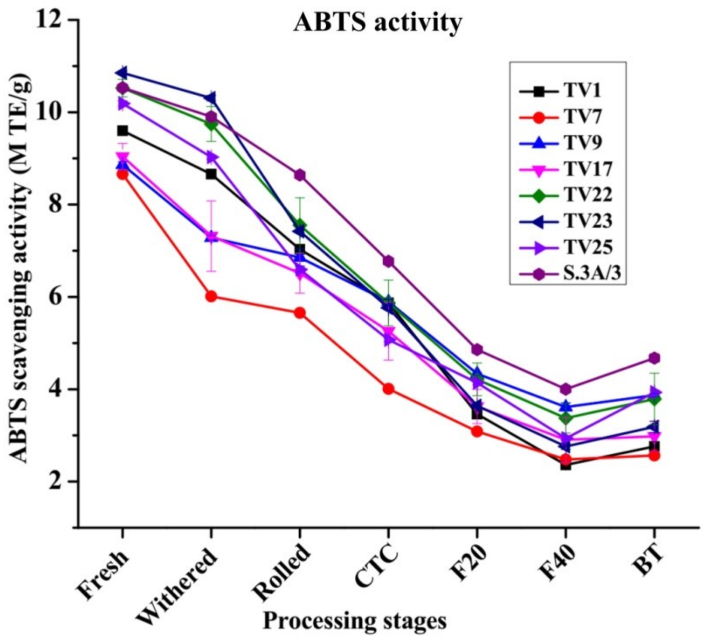

Fig. S1. Changes in antioxidant activity from fresh leaves to black tea during processing: (b) ABTS scavenging activity. F20, 20 minutes of fermentation; F40, 40 minutes of fermentation.

2. Resonance structure of catechins

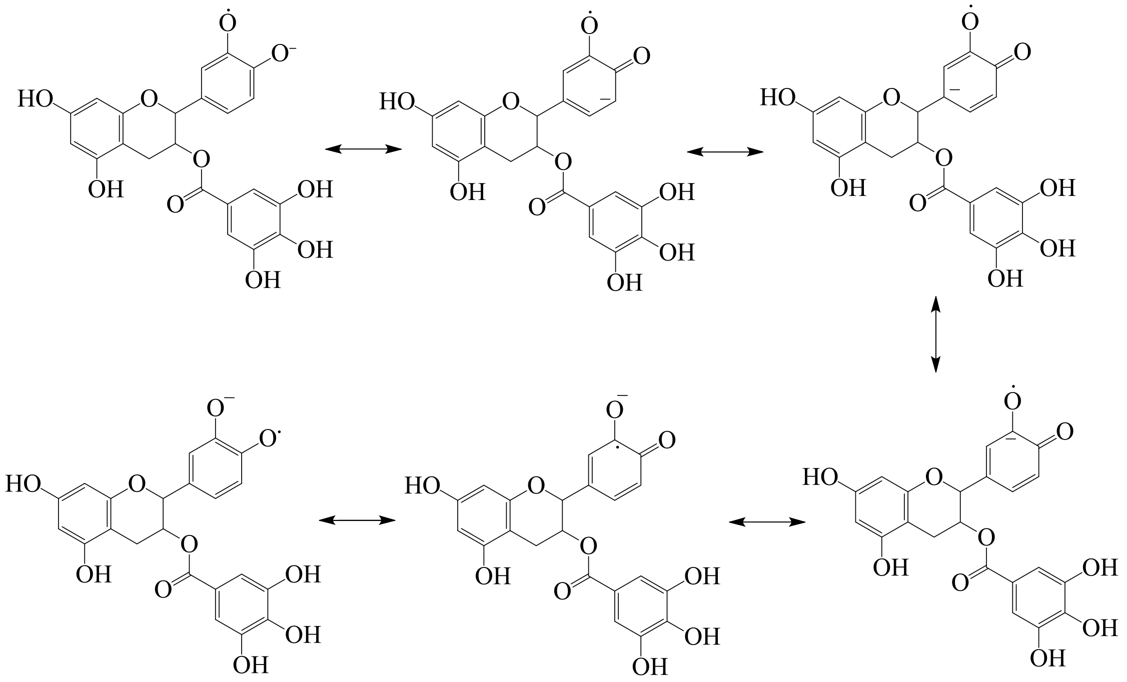

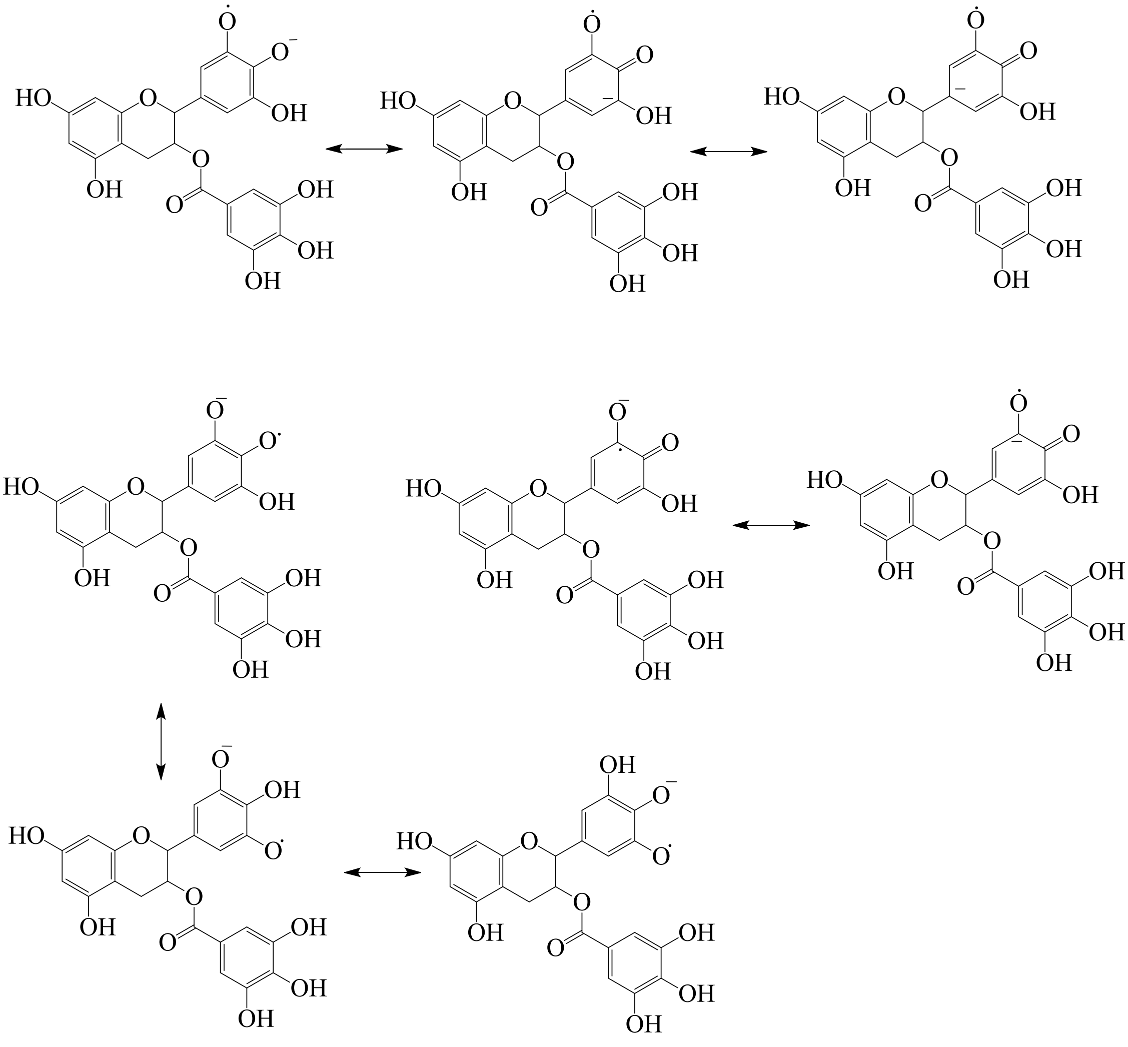

Scheme S1: (a) Resonance structure of trihydroxy catechin (EGCG) semiquinone radical and (b) Resonance structure of dihydroxy catechin (ECG) semiquinone radical.

3. Correlation Study

Table S1A: Correlation between catechins and theaflavins during black tea processing from cultivar TV1

	EGC	EC	EGCG	ECG	TC	Total TF	TF1	TF2A	TF2B	TF3
EGC	1	0.738**	0.894**	0.801**	0.920**	-0.931**	-0.796**	-0.822**	-0.913**	-0.952**
EC		1	0.887**	0.897**	0.904**	-0.579*	-0.393	-0.598**	-0.497*	-0.600**
EGCG			1	0.933**	0.991**	-0.837**	-0.734**	-0.783**	-0.789**	-0.845**
ECG				1	0.955**	-0.725**	-0.629**	-0.696**	-0.669**	-0.729**
TC					1	-0.843**	-0.721**	-0.787**	-0.794**	-0.854**
Total TF						1	0.932**	0.926**	0.954**	0.997**
TF1							1	0.786**	0.956**	0.912**
TF2A								1	0.769**	0.917**
TF2B									1	0.955**
TF3										1

EGC, (−)-epigallocatechin; EC, (−)-epicatechin; EGCG, (−)-epigallocatechin gallate; ECG, (−)-epicatechin gallate; TC, total catechin; Total TF, total theaflavin; TF1, theaflavin; TF2A, theaflavin-3-gallate; TF2B, theaflavin-3'-gallate; TF3, theaflavin-3,3'-digallate. *Significant at the 0.05 level; **Significant at the 0.01 level.

Table S1B: Correlation between catechins and theaflavins during black tea processing from cultivar TV7

	EGC	EC	EGCG	ECG	TC	Total TF	TF1	TF2A	TF2B	TF3
EGC	1	0.969**	0.953**	0.841**	0.959**	-0.876**	0.165	-0.629**	-0.889**	-0.966**
EC		1	0.939**	0.841**	0.948**	-0.783**	0.316	-0.599**	-0.845**	-0.920**
EGCG			1	0.953**	0.999**	-0.826**	0.214	-0.604**	-0.834**	-0.925**
ECG				1	0.955**	-0.725**	0.208	-0.587*	-0.690**	-0.807**
TC					1	-0.828**	0.215	-0.617**	-0.832**	-0.927**
Total TF						1	0.262	0.776**	0.898**	0.950**
TF1							1	0.105	0.107	-0.039
TF2A								1	0.541*	0.724**
TF2B									1	0.931**
TF3										1

EGC, (−)-epigallocatechin; EC, (−)-epicatechin; EGCG, (−)-epigallocatechin gallate; ECG, (−)-epicatechin gallate; TC, total catechin; Total TF, total theaflavin; TF1, theaflavin; TF2A, theaflavin-3-gallate; TF2B, theaflavin-3'-gallate; TF3, theaflavin-3,3'-digallate. *Significant at the 0.05 level; **Significant at the 0.01 level.

Table S1C: Correlation between catechins and theaflavins during black tea processing from cultivar TV9

	EGC	EC	EGCG	ECG	TC	Total TF	TF1	TF2A	TF2B	TF3
EGC	1	0.976**	0.942**	0.966**	0.974**	-0.907**	-0.943**	-0.798**	-0.899**	-0.816**
EC		1	0.954**	0.962**	0.976**	-0.927**	-0.935**	-0.825**	-0.906**	-0.840**
EGCG			1	.966**	0.993**	-0.955**	-0.890**	-0.886**	-0.914**	-0.917**
ECG				1	0.985**	-0.908**	-0.880**	-0.817**	-0.893**	-0.866**
TC					1	-0.948**	-0.915**	-0.864**	-0.919**	-0.894**
Total TF						1	0.938**	0.906**	0.963**	0.940**
TF1							1	0.863**	0.907**	0.857**
TF2A								1	0.768**	0.956**
TF2B									1	0.862**
TF3										1

EGC, (−)-epigallocatechin; EC, (−)-epicatechin; EGCG, (−)-epigallocatechin gallate; ECG, (−)-epicatechin gallate; TC, total catechin; Total TF, total theaflavin; TF1, theaflavin; TF2A, theaflavin-3-gallate; TF2B, theaflavin-3'-gallate; TF3, theaflavin-3,3'-digallate. *Significant at the 0.05 level; **Significant at the 0.01 level.

Table S1D: Correlation between catechins and theaflavins during black tea processing from cultivar TV17

	EGC	EC	EGCG	ECG	TC	Total TF	TF1	TF2A	TF2B	TF3
EGC	1	0.797**	0.941**	0.790**	0.923**	-0.769**	-0.722**	-0.785**	-0.757**	-0.784**
EC		1	0.939**	0.995**	0.964**	-0.652**	-0.690**	-0.403	-0.794**	-0.659**
EGCG			1	0.943**	0.995**	-0.785**	-0.794**	-0.659**	-0.851**	-0.802**
ECG				1	0.964**	-0.684**	-0.728**	-0.422	-0.833**	-0.701**
TC					1	-0.759**	-0.768**	-0.604**	-0.844**	-0.774**
Total TF						1	0.953**	0.848**	0.912**	0.972**
TF1							1	0.752**	0.928**	0.930**
TF2A								1	0.641**	0.825**
TF2B									1	0.951**
TF3										1

EGC, (−)-epigallocatechin; EC, (−)-epicatechin; EGCG, (−)-epigallocatechin gallate; ECG, (−)-epicatechin gallate; TC, total catechin; Total TF, total theaflavin; TF1, theaflavin; TF2A, theaflavin-3-gallate; TF2B, theaflavin-3'-gallate; TF3, theaflavin-3,3'-digallate. *Significant at the 0.05 level; **Significant at the 0.01 level.

Table S1E: Correlation between catechins and theaflavins during black tea processing from cultivar TV22

	EGC	EC	EGCG	ECG	TC	Total TF	TF1	TF2A	TF2B	TF3
EGC	1	0.819**	0.914**	0.784**	0.924**	-0.941**	-0.692**	-0.772**	-0.935**	-0.957**
EC		1	0.946**	0.958**	0.957**	-0.861**	-0.644**	-0.631**	-0.923**	-0.874**
EGCG			1	0.953**	0.997**	-0.936**	-0.731**	-0.750**	-0.955**	-0.947**
ECG				1	0.957**	-0.850**	-0.640**	-0.626**	-0.908**	-0.864**
TC					1	-0.941**	-0.716**	-0.739**	-0.968**	-0.954**
Total TF						1	0.841**	0.889**	0.966**	0.987**
TF1							1	0.961**	0.704**	0.749**
TF2A								1	0.743**	0.816**
TF2B									1	0.985**
TF3										1

EGC, (−)-epigallocatechin; EC, (−)-epicatechin; EGCG, (−)-epigallocatechin gallate; ECG, (−)-epicatechin gallate; TC, total catechin; Total TF, total theaflavin; TF1, theaflavin; TF2A, theaflavin-3-gallate; TF2B, theaflavin-3'-gallate; TF3, theaflavin-3,3'-digallate. *Significant at the 0.05 level; **Significant at the 0.01 level.

Table S1F: Correlation between catechins and theaflavins during black tea processing from cultivar TV23

	EGC	EC	EGCG	ECG	TC	Total TF	TF1	TF2A	TF2B	TF3
EGC	1	0.895**	0.983**	0.927**	0.988**	-0.944**	-0.834**	-0.812**	-0.951**	-0.946**
EC		1	0.908**	0.965**	0.941**	-0.850**	-0.665**	-0.644**	-0.912**	-0.883**
EGCG			1	0.924**	0.993**	-0.919**	-0.762**	-0.740**	-0.954**	-0.941**
ECG				1	0.959**	-0.886**	-0.697**	-0.696**	-0.941**	-0.901**
TC					1	-0.931**	-0.775**	-0.755**	-0.965**	-0.948**
Total TF						1	0.889**	0.917**	0.969**	0.988**
TF1							1	0.977**	0.766**	0.821**
TF2A								1	0.793**	0.851**
TF2B									1	0.990**
TF3										1

EGC, (−)-epigallocatechin; EC, (−)-epicatechin; EGCG, (−)-epigallocatechin gallate; ECG, (−)-epicatechin gallate; TC, total catechin; Total TF, total theaflavin; TF1, theaflavin; TF2A, theaflavin-3-gallate; TF2B, theaflavin-3'-gallate; TF3, theaflavin-3,3'-digallate. *Significant at the 0.05 level; **Significant at the 0.01 level.

Table S1G: Correlation between catechins and theaflavins during black tea processing from cultivar TV25

	EGC	EC	EGCG	ECG	TC	Total TF	TF1	TF2A	TF2B	TF3
EGC	1	0.907**	0.960**	0.853**	0.969**	-0.920**	-0.847**	-0.813**	-0.879**	-0.917**
EC		1	0.977**	0.972**	0.977**	-0.923**	-0.808**	-0.648**	-0.943**	-0.948**
EGCG			1	0.958**	0.999**	-0.933**	-0.803**	-0.696**	-0.938**	-0.953**
ECG				1	0.953**	-0.878**	-0.712**	-0.546*	-0.922**	-0.919**
TC					1	-0.938**	-0.819**	-0.717**	-0.937**	-0.955**
Total TF						1	0.935**	0.835**	0.979**	0.994**
TF1							1	0.900**	0.871**	0.898**
TF2A								1	0.705**	0.774**
TF2B									1	0.993**
TF3										1

EGC, (−)-epigallocatechin; EC, (−)-epicatechin; EGCG, (−)-epigallocatechin gallate; ECG, (−)-epicatechin gallate; TC, total catechin; Total TF, total theaflavin; TF1, theaflavin; TF2A, theaflavin-3-gallate; TF2B, theaflavin-3'-gallate; TF3, theaflavin-3,3'-digallate. *Significant at the 0.05 level; **Significant at the 0.01 level.

Table S1H: Correlation between catechins and theaflavins during black tea processing from cultivar S.3A/3

	EGC	EC	EGCG	ECG	TC	Total TF	TF1	TF2A	TF2B	TF3
EGC	1	0.805**	0.879**	0.700**	0.898**	-0.855**	-0.834**	-0.734**	-0.835**	-0.856**
EC		1	0.942**	0.924**	0.950**	-0.950**	-0.810**	-0.735**	-0.970**	-0.963**
EGCG			1	0.940**	0.997**	-0.954**	-0.802**	-0.779**	-0.951**	-0.966**
ECG				1	0.936**	-0.869**	-0.619**	-0.617**	-0.904**	-0.901**
TC					1	-0.953**	-0.804**	-0.765**	-0.955**	-0.967**
Total TF						1	0.919**	0.874**	0.981**	0.996**
TF1							1	0.887**	0.873**	0.890**
TF2A								1	0.764**	0.833**
TF2B									1	0.991**
TF3										1

EGC, (−)-epigallocatechin; EC, (−)-epicatechin; EGCG, (−)-epigallocatechin gallate; ECG, (−)-epicatechin gallate; TC, total catechin; Total TF, total theaflavin; TF1, theaflavin; TF2A, theaflavin-3-gallate; TF2B, theaflavin-3'-gallate; TF3, theaflavin-3,3'-digallate. *Significant at the 0.05 level; **Significant at the 0.01 level.

Table S2A: Correlation between catechins, total polyphenol and antioxidant activity during black tea processing from cultivar TV1

	EGC	+C	EC	EGCG	ECG	TC	TP	DPPH	ABTS	FRAP
EGC	1	0.567**	0.752**	0.930**	0.843**	0.945**	0.545*	0.737**	0.905**	0.866**
+C		1	0.354	0.619**	0.458*	0.612**	0.279	0.578**	0.593**	0.557**
EC			1	0.854**	0.901**	0.874**	0.858**	0.830**	0.847**	0.869**
EGCG				1	0.932**	0.994**	0.665**	0.848**	0.972**	0.952**
ECG					1	0.950**	0.729**	0.826**	0.960**	0.969**
TC						1	0.684**	0.852**	0.979**	0.962**
TP							1	0.818**	0.669**	0.700**
DPPH								1	0.826**	0.818**
ABTS									1	0.991**
FRAP										1

EGC, (−)-epigallocatechin; +C, (+)-catechin: EC, (−)-epicatechin; EGCG, (−)-epigallocatechin gallate; ECG, (−)-epicatechin gallate; TC, total catechin; TP, total polyphenol; DPPH, DPPH scavenging activity; ABTS, ABTS scavenging activity; FRAP, Ferric reducing antioxidant potential. *Significant at the 0.05 level; **Significant at the 0.01 level.

Table S2B: Correlation between catechins, total polyphenol and antioxidant activity during black tea processing from cultivar TV7

	EGC	+C	EC	EGCG	ECG	TC	TP	DPPH	ABTS	FRAP
EGC	1	0.573**	0.942**	0.969**	0.892**	0.973**	0.811**	0.824**	0.917**	0.922**
+C		1	0.484*	0.616**	0.538*	0.622**	0.540**	0.548**	0.770**	0.662**
EC			1	0.919**	0.845**	0.925**	0.805**	0.780**	0.863**	0.884**
EGCG				1	0.963**	0.999**	0.902**	0.908**	0.967**	0.980**
ECG					1	0.964**	0.913**	0.929**	0.934**	0.968**
TC						1	0.897**	0.904**	0.969**	0.981**
TP							1	0.905**	0.963**	0.950**
DPPH								1	0.915**	0.909**
ABTS									1	0.982**
FRAP										1

EGC, (−)-epigallocatechin; +C, (+)-catechin: EC, (−)-epicatechin; EGCG, (−)-epigallocatechin gallate; ECG, (−)-epicatechin gallate; TC, total catechin; TP, total polyphenol; DPPH, DPPH scavenging activity; ABTS, ABTS scavenging activity; FRAP, Ferric reducing antioxidant potential. *Significant at the 0.05 level; **Significant at the 0.01 level.

Table S2C: Correlation between catechins, total polyphenol and antioxidant activity during black tea processing from cultivar TV9

	EGC	+C	EC	EGCG	ECG	TC	TP	DPPH	ABTS	FRAP
EGC	1	0.551**	0.966**	0.960**	0.974**	0.982**	0.937**	0.870**	0.960**	0.962**
+C		1	0.626**	0.565**	0.527*	0.575**	0.551**	0.664**	0.676**	0.688**
EC			1	0.955**	0.951**	0.971**	0.946**	0.917**	0.971**	0.978**
EGCG				1	0.974**	0.995**	0.932**	0.860**	0.979**	0.968**
ECG					1	0.987**	0.947**	0.866**	0.971**	0.962**
TC						1	0.947**	0.878**	0.985**	0.979**
TP							1	0.912**	0.937**	0.935**
DPPH								1	0.891**	0.903**
ABTS									1	0.996**
FRAP										1

EGC, (−)-epigallocatechin; +C, (+)-catechin: EC, (−)-epicatechin; EGCG, (−)-epigallocatechin gallate; ECG, (−)-epicatechin gallate; TC, total catechin; TP, total polyphenol; DPPH, DPPH scavenging activity; ABTS, ABTS scavenging activity; FRAP, Ferric reducing antioxidant potential. *Significant at the 0.05 level; **Significant at the 0.01 level.

Table S2D: Correlation between catechins, total polyphenol and antioxidant activity during black tea processing from cultivar TV17

	EGC	+C	EC	EGCG	ECG	TC	TP	DPPH	ABTS	FRAP
EGC	1	0.622**	0.844**	0.959**	0.808**	0.948**	0.855**	0.849**	0.896**	0.859**
+C		1	0.465*	0.589**	0.391	0.565**	0.558**	0.708**	0.641**	0.572**
EC			1	0.944**	0.984**	0.967**	0.923**	0.887**	0.904**	0.903**
EGCG				1	0.912**	0.993**	0.919**	0.904**	0.927**	0.906**
ECG					1	0.946**	0.879**	0.837**	0.873**	0.863**
TC						1	0.925**	0.907**	0.935**	0.913**
TP							1	0.846**	0.908**	0.909**
DPPH								1	0.893**	0.878**
ABTS									1	0.987**
FRAP										1

EGC, (−)-epigallocatechin; +C, (+)-catechin: EC, (−)-epicatechin; EGCG, (−)-epigallocatechin gallate; ECG, (−)-epicatechin gallate; TC, total catechin; TP, total polyphenol; DPPH, DPPH scavenging activity; ABTS, ABTS scavenging activity; FRAP, Ferric reducing antioxidant potential. *Significant at the 0.05 level; **Significant at the 0.01 level.

Table S2E: Correlation between catechins, total polyphenol and antioxidant activity during black tea processing from cultivar TV22

	EGC	+C	EC	EGCG	ECG	TC	TP	DPPH	ABTS	FRAP
EGC	1	0.655**	0.860**	0.943**	0.838**	0.950**	0.855**	0.767**	0.943**	0.912**
+C		1	0.490*	0.622**	0.514*	0.616**	0.612**	0.575**	0.604**	0.581**
EC			1	0.948**	0.960**	0.957**	0.958**	0.780**	0.934**	0.936**
EGCG				1	0.961**	0.998**	0.956**	0.855**	0.969**	0.938**
ECG					1	0.963**	0.966**	0.806**	0.905**	0.896**
TC						1	0.958**	0.838**	0.970**	0.946**
TP							1	0.784**	0.930**	0.936**
DPPH								1	0.850**	0.759**
ABTS									1	0.976**
FRAP										1

EGC, (−)-epigallocatechin; +C, (+)-catechin: EC, (−)-epicatechin; EGCG, (−)-epigallocatechin gallate; ECG, (−)-epicatechin gallate; TC, total catechin; TP, total polyphenol; DPPH, DPPH scavenging activity; ABTS, ABTS scavenging activity; FRAP, Ferric reducing antioxidant potential. *Significant at the 0.05 level; **Significant at the 0.01 level.

Table S2F: Correlation between catechins, total polyphenol and antioxidant activity during black tea processing from cultivar TV23

	EGC	+C	EC	EGCG	ECG	TC	TP	DPPH	ABTS	FRAP
EGC	1	0.548*	0.925**	0.987**	0.942**	0.989**	0.899**	0.829**	0.960**	0.980**
+C		1	0.649**	0.540*	0.621**	0.597**	0.643**	0.758**	0.599**	0.567**
EC			1	0.930**	0.968**	0.960**	0.957**	0.899**	0.946**	0.944**
EGCG				1	0.943**	0.993**	0.910**	0.832**	0.972**	0.983**
ECG					1	0.971**	0.981**	0.869**	0.933**	0.943**
TC						1	0.941**	0.865**	0.975**	0.986**
TP							1	0.881**	0.908**	0.911**
DPPH								1	0.937**	0.957**
ABTS									1	0.992**
FRAP										1

EGC, (−)-epigallocatechin; +C, (+)-catechin: EC, (−)-epicatechin; EGCG, (−)-epigallocatechin gallate; ECG, (−)-epicatechin gallate; TC, total catechin; TP, total polyphenol; DPPH, DPPH scavenging activity; ABTS, ABTS scavenging activity; FRAP, Ferric reducing antioxidant potential. *Significant at the 0.05 level; **Significant at the 0.01 level.

Table S2G: Correlation between catechins, total polyphenol and antioxidant activity during black tea processing from cultivar TV25

	EGC	+C	EC	EGCG	ECG	TC	TP	DPPH	ABTS	FRAP
EGC	1	0.712**	0.946**	0.978**	0.913**	0.985**	0.934**	0.807**	0.923**	0.905**
+C		1	0.654**	0.664**	0.616**	0.697**	0.664**	0.538*	0.570**	0.577**
EC			1	0.979**	0.973**	0.981**	0.955**	0.858**	0.964**	0.954**
EGCG				1	0.971**	0.998**	0.965**	0.862**	0.962**	0.952**
ECG					1	0.967**	0.960**	0.893**	0.967**	0.961**
TC						1	0.965**	0.857**	0.959**	0.947**
TP							1	0.871**	0.946**	0.948**
DPPH								1	0.919**	0.937**
ABTS									1	0.993**
FRAP										1

EGC, (−)-epigallocatechin; +C, (+)-catechin: EC, (−)-epicatechin; EGCG, (−)-epigallocatechin gallate; ECG, (−)-epicatechin gallate; TC, total catechin; TP, total polyphenol; DPPH, DPPH scavenging activity; ABTS, ABTS scavenging activity; FRAP, Ferric reducing antioxidant potential. *Significant at the 0.05 level; **Significant at the 0.01 level.

Table S2H: Correlation between catechins, total polyphenol and antioxidant activity during black tea processing from cultivar S.3A/3

	EGC	+C	EC	EGCG	ECG	TC	TP	DPPH	ABTS	FRAP
EGC	1	0.676**	0.866**	0.932**	0.785**	0.944**	0.805**	0.903**	0.953**	0.933**
+C		1	0.547*	0.651**	0.519*	0.655**	0.739**	0.755**	0.669**	0.637**
EC			1	0.942**	0.929**	0.951**	0.857**	0.876**	0.911**	0.944**
EGCG				1	0.937**	0.998**	0.918**	0.934**	0.978**	0.987**
ECG					1	0.938**	0.898**	0.862**	0.899**	0.934**
TC						1	0.914**	0.939**	0.982**	0.990**
TP							1	0.882**	0.884**	0.893**
DPPH								1	0.962**	0.956**
ABTS									1	0.993**
FRAP										1

EGC, (−)-epigallocatechin; +C, (+)-catechin: EC, (−)-epicatechin; EGCG, (−)-epigallocatechin gallate; ECG, (−)-epicatechin gallate; TC, total catechin; TP, total polyphenol; DPPH, DPPH scavenging activity; ABTS, ABTS scavenging activity; FRAP, Ferric reducing antioxidant potential. *Significant at the 0.05 level; **Significant at the 0.01 level.

Table S3A: Correlation between theaflavins, total polyphenol and antioxidant activity during black tea processing from cultivar TV1

	Total TF	TF1	TF2A	TF2B	TF3	TP	DPPH	ABTS	FRAP
Total TF	1	0.932**	0.926**	0.954**	0.997**	-0.412	-0.512*	-0.780**	-0.719**
TF1		1	0.786**	0.956**	0.912**	-0.282	-0.427	-0.688**	-0.615**
TF2A			1	0.769**	0.917**	-0.385	-0.482*	-0.724**	-0.687**
TF2B				1	0.955**	-0.383	-0.478*	-0.738**	-0.665**
TF3					1	-0.434	-0.521*	-0.787**	-0.728**
TP						1	0.874**	0.754**	0.767**
DPPH							1	0.747**	0.745**
ABTS								1	0.992**
FRAP									1

Total TF, total theaflavin; TF1, theaflavin; TF2A, theaflavin-3-gallate; TF2B, theaflavin-3'-gallate; TF3, theaflavin-3,3'-digallate; TP, total polyphenol; DPPH, DPPH scavenging activity; ABTS, ABTS scavenging activity; FRAP, Ferric reducing antioxidant potential. *Significant at the 0.05 level; **Significant at the 0.01 level.

Table S3B: Correlation between theaflavins, total polyphenol and antioxidant activity during black tea processing from cultivar TV7

	Total TF	TF1	TF2A	TF2B	TF3	TP	DPPH	ABTS	FRAP
Total TF	1	0.262	0.776**	0.898**	0.950**	-0.507*	-0.675*	-0.732**	-0.713**
TF1		1	0.105	0.107	-0.039	0.359	0.045	0.309	0.246
TF2A			1	0.541*	0.724**	-0.298	-0.438	-0.536*	-0.511*
TF2B				1	0.931**	-0.520*	-0.683**	-0.764**	-0.729**
TF3					1	-0.634**	-0.717**	-0.858**	-0.817**
TP						1	0.878**	0.914**	0.934**
DPPH							1	0.825**	0.937**
ABTS								1	0.991**
FRAP									1

Total TF, total theaflavin; TF1, theaflavin; TF2A, theaflavin-3-gallate; TF2B, theaflavin-3'-gallate; TF3, theaflavin-3,3'-digallate; TP, total polyphenol; DPPH, DPPH scavenging activity; ABTS, ABTS scavenging activity; FRAP, Ferric reducing antioxidant potential. *Significant at the 0.05 level; **Significant at the 0.01 level.

Table S3C: Correlation between theaflavins, total polyphenol and antioxidant activity during black tea processing from cultivar TV9

	Total TF	TF1	TF2A	TF2B	TF3	TP	DPPH	ABTS	FRAP
Total TF	1	0.938**	0.906**	0.963**	0.940**	-0.859**	-0.771**	-0.927**	-0.916**
TF1		1	0.863**	0.907**	0.857**	-0.860**	-0.764**	-0.881**	-0.886**
TF2A			1	0.768**	0.956**	-0.803**	-0.676**	-0.852**	-0.834**
TF2B				1	0.862**	-0.813**	-0.750**	-0.888**	-0.878**
TF3					1	-0.799**	-0.688**	-0.874**	-0.845**
TP						1	0.872**	0.919**	0.917**
DPPH							1	0.803**	0.821**
ABTS								1	0.993**
FRAP									1

Total TF, total theaflavin; TF1, theaflavin; TF2A, theaflavin-3-gallate; TF2B, theaflavin-3'-gallate; TF3, theaflavin-3,3'-digallate; TP, total polyphenol; DPPH, DPPH scavenging activity; ABTS, ABTS scavenging activity; FRAP, Ferric reducing antioxidant potential. *Significant at the 0.05 level; **Significant at the 0.01 level.

Table S3D: Correlation between theaflavins, total polyphenol and antioxidant activity during black tea processing from cultivar TV17

	Total TF	TF1	TF2A	TF2B	TF3	TP	DPPH	ABTS	FRAP
Total TF	1	0.953**	0.848**	0.912**	0.972**	-0.610**	-0.600**	-0.686**	-0.661**
TF1		1	0.752**	0.928**	0.930**	-0.617**	-0.616**	-0.700**	-0.669**
TF2A			1	0.641**	0.825**	-0.466	-0.392	-0.522*	-0.475*
TF2B				1	0.951**	-0.695**	-0.710**	-0.765**	-0.741**
TF3					1	-0.613**	-0.604**	-0.688**	-0.657**
TP						1	0.769**	0.872**	0.869**
DPPH							1	0.832**	0.835**
ABTS								1	0.992**
FRAP									1

Total TF, total theaflavin; TF1, theaflavin; TF2A, theaflavin-3-gallate; TF2B, theaflavin-3'-gallate; TF3, theaflavin-3,3'-digallate; TP, total polyphenol; DPPH, DPPH scavenging activity; ABTS, ABTS scavenging activity; FRAP, Ferric reducing antioxidant potential. *Significant at the 0.05 level; **Significant at the 0.01 level.

Table S3E: Correlation between theaflavins, total polyphenol and antioxidant activity during black tea processing from cultivar **T**V22

	Total TF	TF1	TF2A	TF2B	TF3	TP	DPPH	ABTS	FRAP
Total TF	1	0.841**	0.889**	0.966**	0.987**	-0.812**	-0.642**	-0.900**	-0.895**
TF1		1	0.961**	0.704**	0.749**	-0.667**	-0.510*	-0.702**	-0.727**
TF2A			1	0.743**	0.816**	-0.630**	-0.483*	-0.728**	-0.745**
TF2B				1	0.985**	-0.854**	-0.677**	-0.915**	-0.904**
TF3					1	-0.810**	-0.652**	-0.913**	-0.895**
TP						1	0.680**	0.893**	0.927**
DPPH							1	0.806**	0.690**
ABTS								1	0.971**
FRAP									1

Total TF, total theaflavin; TF1, theaflavin; TF2A, theaflavin-3-gallate; TF2B, theaflavin-3'-gallate; TF3, theaflavin-3,3'-digallate; TP, total polyphenol; DPPH, DPPH scavenging activity; ABTS, ABTS scavenging activity; FRAP, Ferric reducing antioxidant potential. *Significant at the 0.05 level; **Significant at the 0.01 level.

Table S3F: Correlation between theaflavins, total polyphenol and antioxidant activity during black tea processing from cultivar TV23

	Total TF	TF1	TF2A	TF2B	TF3	TP	DPPH	ABTS	FRAP
Total TF	1	0.889**	0.917**	0.969**	0.988**	-0.782**	-0.662**	-0.890**	-0.925**
TF1		1	0.977**	0.766**	0.821**	-0.607**	-0.542*	-0.754**	-0.804**
TF2A			1	0.793**	0.851**	-0.579*	-0.467	-0.721**	-0.774**
TF2B				1	0.990**	-0.854**	-0.734**	-0.917**	-0.942**
TF3					1	-0.806**	-0.687**	-0.920**	-0.942**
TP						1	0.839**	0.858**	0.875**
DPPH							1	0.908**	0.940**
ABTS								1	0.991**
FRAP									1

Total TF, total theaflavin; TF1, theaflavin; TF2A, theaflavin-3-gallate; TF2B, theaflavin-3'-gallate; TF3, theaflavin-3,3'-digallate; TP, total polyphenol; DPPH, DPPH scavenging activity; ABTS, ABTS scavenging activity; FRAP, Ferric reducing antioxidant potential. *Significant at the 0.05 level; **Significant at the 0.01 level.

Table S3G: Correlation between theaflavins, total polyphenol and antioxidant activity during black tea processing from cultivar TV25

	Total TF	TF1	TF2A	TF2B	TF3	TP	DPPH	ABTS	FRAP
Total TF	1	0.935**	0.835**	0.979**	0.994**	-0.813**	-0.806**	-0.916**	-0.877**
TF1		1	0.900**	0.871**	0.898**	-0.668**	-0.611**	-0.785**	-0.731**
TF2A			1	0.705**	0.774**	-0.562*	-0.437	-0.618**	-0.551*
TF2B				1	0.993**	-0.830**	-0.875**	-0.948**	-0.923**
TF3					1	-0.845**	-0.850**	-0.944**	-0.911**
TP						1	0.841**	0.933**	0.934**
DPPH							1	0.902**	0.924**
ABTS								1	0.991**
FRAP									1

Total TF, total theaflavin; TF1, theaflavin; TF2A, theaflavin-3-gallate; TF2B, theaflavin-3'-gallate; TF3, theaflavin-3,3'-digallate; TP, total polyphenol; DPPH, DPPH scavenging activity; ABTS, ABTS scavenging activity; FRAP, Ferric reducing antioxidant potential. *Significant at the 0.05 level; **Significant at the 0.01 level.

Table S3H: Correlation between theaflavins, total polyphenol and antioxidant activity during black tea processing from cultivar S.3A/3

	Total TF	TF1	TF2A	TF2B	TF3	TP	DPPH	ABTS	FRAP
Total TF	1	0.919**	0.874**	0.981**	0.996**	-0.788**	-0.841**	-0.886**	-0.927**
TF1		1	0.887**	0.873**	0.890**	-0.608**	-0.682**	-0.747**	-0.771**
TF2A			1	0.764**	0.833**	-0.569*	-0.713**	-0.750**	-0.767**
TF2B				1	0.991**	-0.820**	-0.824**	-0.870**	-0.919**
TF3					1	-0.809**	-0.854**	-0.897**	-0.939**
TP						1	0.734**	0.776**	0.811**
DPPH							1	0.951**	0.952**
ABTS								1	0.989**
FRAP									1

Total TF, total theaflavin; TF1, theaflavin; TF2A, theaflavin-3-gallate; TF2B, theaflavin-3'-gallate; TF3, theaflavin-3,3'-digallate; TP, total polyphenol; DPPH, DPPH scavenging activity; ABTS, ABTS scavenging activity; FRAP, Ferric reducing antioxidant potential. *Significant at the 0.05 level; **Significant at the 0.01 level.

Table S4A Change in total polyphenol (TP) from fresh leaves to black tea during processing

Processing Stages	TV1	TV7	TV9	TV17	TV22	TV23	TV25	S.3A/3
Fresh	199.3±9.8	196.1±4.2	202.9±4.8	246.4±7.4	248.1±3.3	245.3±4.1	222.5±4.0	239.2±2.6
Withered	190.9±10.4	185.3±4.9	185.6±4.6	233.7±7.8	221.8±8.4	212.5±7.4	207.5±4.9	199.3±11.2
Rolled	187.6±11.5	181.7±3.3	178.3±6.8	218.4±2.4	211.7±7.1	208.0±9.0	178.5±5.3	191.8±12.3
CTC	180.9±13.1	169.8±3.1	173.6±5.2	203.6±13.4	203.7±10.4	199.1±7.7	172.4±2.6	188.5±4.6
F20	167.6±6.6	161.3±3.4	137.4±6.5	184.3±6.1	172.0±7.7	177.4±3.4	155.8±4.2	163.3±6.7
F40	136.9±16.6	132.5±1.8	123.3±8.4	162.4±5.5	153.4±6.0	150.0±2.7	143.9±2.5	156.2±4.2
BT	148.1±8.2	138.5±1.2	133.6±6.7	167.6±8.1	158.8±5.6	155.2±1.4	154.0±3.0	150.0±1.3

Fresh, fresh leaves; Withered, withered leaves; Rolled, rolled leaves; CTC, CTC leaves ; F20, 20 minutes of fermentation; F40, 40 minutes of fermentation; BT, black tea. All units are in mg g^{-1} (dry weight basis). Values are 'mean±SE' of independent triplicate measurements.

Table S4B Change in total epigallocatechingallate (EGCG) from fresh leaves to black tea during processing

Processing Stages	TV1	TV7	TV9	TV17	TV22	TV23	TV25	S.3A/3
Fresh	66.7±1.6	71.0±4.2	97.8±1.8	63.5±6.5	89.5±5.0	93.7±4.1	72.6±6.5	105.7±5.6
Withered	53.9±7.5	60.0±0.8	86.5±4.2	54.8±1.1	72.8±5.6	85.2±3.4	56.6±2.1	85.5±4.3
Rolled	42.4±1.5	45.2±1.8	72.5±2.4	45.6±1.1	53.8±3.8	71.3±5.5	37.9±1.5	58.2±2.6
CTC	23.5±5.6	18.3±1.0	43.6±0.5	19.2±1.2	30.6±6.9	45.7±4.0	18.2±1.0	40.2±1.5
F20	5.0±0.3	6.2±0.4	11.4±0.6	4.5±0.3	9.3±1.9	14.5±2.6	8.4±0.3	11.8±0.5
F40	2.1±0.2	2.2±0.2	4.5±0.4	1.9±0.1	3.9±0.9	6.5±0.5	4.1±0.7	4.7±0.4
BT	1.7±0.1	1.5±0.2	1.8±0.1	1.6±0.2	1.8±0.2	2.8±0.4	1.9±0.2	2.8±0.7

Fresh, fresh leaves; Withered, withered leaves; Rolled, rolled leaves; CTC, CTC leaves ; F20, 20 minutes of fermentation; F40, 40 minutes of fermentation; BT, black tea. All units are in mg g^{-1} (dry weight basis). Values are 'mean±SE' of independent triplicate measurements.

Table S4C Change in total epicatechingallate (ECG) from fresh leaves to black tea during processing

Processing Stages	TV1	TV7	TV9	TV17	TV22	TV23	TV25	S.3A/3
Fresh	29.5±1.6	25.9±1.9	30.9±0.8	46.3±0.8	45.0±3.6	38.0±1.3	27.9±1.1	34.8±1.6
Withered	28.9±1.2	23.0±1.4	27.3±0.7	45.9±1.4	39.4±4.1	28.0±1.5	23.7±0.6	32.6±1.4
Rolled	24.4±2.3	18.3±1.2	23.9±1.5	45.6±2.5	33.8±2.5	26.9±2.0	17.3±1.2	26.2±0.3
CTC	20.9±2.7	14.2±0.9	20.7±0.2	34.0±0.9	28.2±3.4	21.0±1.6	14.9±0.7	24.0±1.7
F20	7.3±0.6	5.6±0.4	8.5±0.5	14.1±0.8	14.6±2.8	10.4±1.4	7.1±0.6	13.4±0.4
F40	3.2±0.3	3.5±0.2	3.2±0.4	6.1±0.5	7.0±2.0	3.4±0.3	5.7±0.5	5.1±0.1
BT	3.1±0.9	1.4±0.1	2.0±0.3	2.8±0.2	3.5±1.7	2.7±0.2	2.8±0.1	4.3±1.1

Fresh, fresh leaves; Withered, withered leaves; Rolled, rolled leaves; CTC, CTC leaves ; F20, 20 minutes of fermentation; F40, 40 minutes of fermentation; BT, black tea. All units are in mg g^{-1} (dry weight basis). Values are 'mean±SE' of independent triplicate measurements.

Table S4D Change in epigallocatechin (EGC) from fresh leaves to black tea during processing

Processing Stages	TV1	TV7	TV9	TV17	TV22	TV23	TV25	S.3A/3
Fresh	26.8±0.7	18.5±1.5	36.4±1.1	46.5±2.1	42.5±1.6	43.8±2.3	50.5±3.4	36.9±1.1
Withered	22.6±0.5	17.1±1.5	27.8±0.5	40.3±2.8	33.2±3.0	37.3±1.9	36.5±0.6	26.7±3.9
Rolled	21.8±1.1	11.6±1.1	26.6±1.2	26.6±5.2	25.1±3.4	33.7±2.8	20.6±0.9	20.7±1.1
CTC	ND	ND	24.6±0.6	ND	ND	21.5±2.4	ND	ND
F20	ND	ND	ND	ND	ND	ND	ND	ND
F40	ND	ND	ND	ND	ND	ND	ND	ND
BT	ND	ND	ND	ND	ND	ND	ND	ND

Fresh, fresh leaves; Withered, withered leaves; Rolled, rolled leaves; CTC, CTC leaves ; F20, 20 minutes of fermentation; F40, 40 minutes of fermentation; BT, black tea; ND, not detected. All units are in mg g^{-1} (dry weight basis). Values are 'mean±SE' of independent triplicate measurements.

Table S4E Change in epicatechin (EC) from fresh leaves to black tea during processing

Processing Stages	TV1	TV7	TV9	TV17	TV22	TV23	TV25	S.3A/3
Fresh	10.4±2.0	5.7±0.8	10.3±0.9	30.1±1.8	18.1±1.3	22.0±1.5	16.9±2.1	10.6±1.0
Withered	9.9±1.2	5.8±0.7	6.7±0.5	27.7±0.4	16.3±1.4	16.3±0.4	12.7±0.5	8.5±0.7
Rolled	10.5±0.9	5.3±0.8	6.5±0.9	26.7±1.3	14.2±1.2	12.8±2.1	8.8±0.6	7.2±0.3
CTC	7.3±1.9	ND	5.2±0.3	19.6±0.7	10.6±2.1	9.9±2.1	5.5±0.5	6.1±0.6
F20	5.6±1.0	ND	ND	ND	5.3±0.4	5.0±0.4	ND	ND
F40	ND	ND	ND	ND	ND	ND	ND	ND
BT	ND	ND	ND	ND	ND	ND	ND	ND

Fresh, fresh leaves; Withered, withered leaves; Rolled, rolled leaves; CTC, CTC leaves ; F20, 20 minutes of fermentation; F40, 40 minutes of fermentation; BT, black tea; ND, not detected. All units are in mg g^{-1} (dry weight basis). Values are 'mean±SE' of independent triplicate measurements.

Table S4F Change in total catechin (TC) from fresh leaves to black tea during processing

Processing Stages	TV1	TV7	TV9	TV17	TV22	TV23	TV25	S.3A/3
Fresh	143.0±3.5	126.8±6.2	178.1±2.5	190.5±8.4	197.5±7.6	204.1±6.6	173.7±12.1	191.5±5.3
Withered	115.3±7.8	105.9±2.2	148.3±3.7	169.1±3.5	161.6±11.8	165.7±3.0	129.5±2.8	153.3±5.2
Rolled	99.1±5.3	80.4±3.3	129.5±5.0	144.8±7.8	127.0±9.7	145.8±5.6	84.6±2.0	110.1±3.6
CTC	51.7±10.1	32.5±1.9	94.2±0.9	72.7±2.6	69.5±12.3	98.0±1.6	38.6±1.3	72.5±1.9
F20	17.9±1.5	11.8±0.8	19.9±1.2	26.3±1.5	29.1±4.7	29.9±1.3	15.5±0.6	25.1±0.7
F40	5.3±0.5	5.6±0.4	7.6±0.8	8.0±0.5	11.0±2.7	9.9±0.7	9.7±1.1	9.7±0.4
BT	3.1±0.9	2.9±0.3	3.8±0.2	3.9±0.7	5.3±1.7	5.5±0.6	4.7±0.3	7.1±1.4

Fresh, fresh leaves; Withered, withered leaves; Rolled, rolled leaves; CTC, CTC leaves ; F20, 20 minutes of fermentation; F40, 40 minutes of fermentation; BT, black tea. All units are in mg g^{-1} (dry weight basis). Values are 'mean±SE' of independent triplicate measurements.

Table S5A Change in total theaflavin from fresh leaves to black tea during processing

Processing Stages	TV1	TV7	TV9	TV17	TV22	TV23	TV25	S.3A/3
Fresh	ND	ND	ND	ND	ND	ND	ND	ND
Withered	4.2±0.1	4.0±0.2	4.3±0.2	5.1±0.1	4.0±0.2	4.6±0.3	3.2±0.3	4.0±0.1
Rolled	6.2±0.3	7.1±0.2	4.9±0.4	8.9±0.2	6.0±0.5	5.4±0.4	4.3±0.2	6.9±0.2
CTC	15.2±0.4	8.2±0.2	8.2±0.1	9.7±0.2	11.2±0.6	10.1±0.1	9.0±0.5	10.1±0.3
F20	18.1±0.2	9.2±0.1	11.7±0.2	14.4±0.3	15.3±0.7	18.9±0.7	11.4±0.8	17.8±0.2
F40	12.2±0.2	7.6±0.3	10.0±0.5	9.2±0.2	13.3±0.3	14.3±1.1	9.5±0.4	14.3±0.2
BT	15.9±0.4	8.2±0.1	11.5±0.2	12.5±0.3	14.2±0.3	17.9±0.2	11.2±0.4	16.9±0.2

Fresh, fresh leaves; Withered, withered leaves; Rolled, rolled leaves; CTC, CTC leaves ; F20, 20 minutes of fermentation; F40, 40 minutes of fermentation; BT, black tea; ND, not detected. All units are in mg g^{-1} (dry weight basis). Values are 'mean±SE' of independent triplicate measurements.

Table S5B Change in theaflavin (TF1) from fresh leaves to black tea during processing

Processing Stages	TV1	TV7	TV9	TV17	TV22	TV23	TV25	S.3A/3
Fresh	ND	ND	ND	ND	ND	ND	ND	ND
Withered	0.9±0	0.8±0	ND	ND	0.3±0	0.3±0	ND	ND
Rolled	1.2±0.1	1.3±0	0.2±0	0.4±0	0.0±0	0.0±0	ND	ND
CTC	1.6±0	0.9±0	0.3±0	0.3±0	0.4±0	0.4±0	0.2±0	0.3±0
F20	2.2±0	1.1±0	0.7±0	0.9±0	0.8±0	1.0±0	0.3±0	0.5±0
F40	1.5±0	0.9±0	0.5±0	0.5±0	0.6±0	0.6±0	0.2±0	0.3±0
BT	1.5±0	0.8±0	0.6±0	0.6±0	0.4±0	0.6±0	0.2±0	0.3±0

Fresh, fresh leaves; Withered, withered leaves; Rolled, rolled leaves; CTC, CTC leaves ; F20, 20 minutes of fermentation; F40, 40 minutes of fermentation; BT, black tea; ND, not detected. All units are in mg g^{-1} (dry weight basis). Values are 'mean±SE' of independent triplicate measurements.

Table S5C Change in theaflavin-3-gallate (TF2A) from fresh leaves to black tea during processing

Processing Stages	TV1	TV7	TV9	TV17	TV22	TV23	TV25	S.3A/3
Fresh	ND	ND	ND	ND	ND	ND	ND	ND
Withered	2.2±0.1	2.0±0.1	1.4±0.1	1.7±0	2.1±0.1	2.4±0.2	1.5±0.1	1.9±0.1
Rolled	2.7±0.1	3.1±0.1	3.2±0.2	5.8±0.2	2.7±0.2	2.4±0.2	2.1±0.1	3.3±0.1
CTC	4.7±0.1	2.5±0.1	3.8±0.1	4.5±0.1	3.5±0.2	3.2±0	3.1±0.2	3.5±0.1
F20	5.3±0.1	2.7±0	5.1±0.1	6.3±0.1	5.6±0.3	7.0±0.3	3.3±0.2	5.1±0.1
F40	3.5±0	2.2±0.1	4.5±0.2	4.2±0.1	3.9±0.1	4.2±0.3	2.3±0.1	3.5±0
BT	6.1±0.2	3.2±0	4.4±0.1	4.8±0.1	3.7±0.1	4.7±0.1	2.6±0.1	4.0±0

Fresh, fresh leaves; Withered, withered leaves; Rolled, rolled leaves; CTC, CTC leaves ; F20, 20 minutes of fermentation; F40, 40 minutes of fermentation; BT, black tea; ND, not detected. All units are in mg g^{-1} (dry weight basis). Values are 'mean±SE' of independent triplicate measurements.

Table S5D Change in theaflavin-3'-gallate (TF2B) from fresh leaves to black tea during processing

Processing Stages	TV1	TV7	TV9	TV17	TV22	TV23	TV25	S.3A/3
Fresh	ND	ND	ND	ND	ND	ND	ND	ND
Withered	0.5±0	0.5±0	0.8±0	1.0±0	0.7±0	0.8±0	0.7±0.1	0.9±0
Rolled	0.9±0	1.1±0	0.5±0	0.9±0	1.1±0.1	1.0±0.1	0.7±0	1.1±0
CTC	3.5±0.1	1.9±0	1.5±0	1.8±0	2.8±0.1	2.6±0	2.0±0.1	2.2±0.1
F20	4.5±0.1	2.3±0	2.7±0.1	3.3±0.1	3.4±0.2	4.2±0.2	2.9±0.2	4.5±0.1
F40	3.0±0	1.9±0.1	2.1±0.1	1.9±0	3.7±0.1	4.0±0.3	2.7±0.1	4.0±0.1
BT	2.7±0.1	1.4±0	3.1±0.1	3.4±0.1	4.2±0.1	5.3±0.1	3.1±0.1	4.7±0

Fresh, fresh leaves; Withered, withered leaves; Rolled, rolled leaves; CTC, CTC leaves ; F20, 20 minutes of fermentation; F40, 40 minutes of fermentation; BT, black tea; ND, not detected. All units are in mg g^{-1} (dry weight basis). Values are 'mean±SE' of independent triplicate measurements.

Table S5E Change in theaflavin-3,3'-digallate (TF3) from fresh leaves to black tea during processing

Processing Stages	TV1	TV7	TV9	TV17	TV22	TV23	TV25	S.3A/3
Fresh	ND	ND	ND	ND	ND	ND	ND	ND
Withered	0.7±0	0.7±0	2.1±0.1	2.4±0.1	1.0±0.1	1.1±0.1	1.0±0.1	1.2±0
Rolled	1.4±0.1	1.6±0	1.0±0.1	1.8±0	2.2±0.2	2.0±0.1	1.6±0.1	2.5±0.1
CTC	5.5±0.1	2.9±0.1	2.5±0	3.0±0.1	4.5±0.2	4.1±0.1	3.6±0.2	4.1±0.1
F20	6.2±0.1	3.2±0	3.2±0.1	3.9±0.1	5.5±0.2	6.8±0.3	4.9±0.3	7.7±0.1
F40	4.2±0.1	2.6±0.1	2.8±0.1	2.6±0.1	5.1±0.1	5.5±0.4	4.3±0.2	6.6±0.1
BT	5.5±0.2	2.9±0	3.4±0.1	3.7±0.1	5.8±0.1	7.4±0.1	5.2±0.2	7.8±0.1

Fresh, fresh leaves; Withered, withered leaves; Rolled, rolled leaves; CTC, CTC leaves ; F20, 20 minutes of fermentation; F40, 40 minutes of fermentation; BT, black tea; ND, not detected. All units are in mg g^{-1} (dry weight basis). Values are 'mean±SE' of independent triplicate measurements.

Table S6 Change in caffeine from fresh leaves to black tea during processing

Processing Stages	TV1	TV7	TV9	TV17	TV22	TV23	TV25	S.3A/3
Fresh	36.0±2.7	39.8±1.0	45.1±0.3	43.4±1.2	37.0±1.2	42.1±2.1	33.1±0.9	33.3±2.1
Withered	42.6±3.9	43.2±0.3	48.3±1.3	50.0±1.7	39.5±1.2	46.6±2.6	34.1±0.8	36.7±0.8
Rolled	42.9±0.6	45.8±0.7	49.3±1.7	57.9±0.9	40.7±2.5	46.8±4.5	35.2±2.1	35.0±0.1
CTC	45.1±3.2	48.3±0.5	52.9±1.0	56.5±1.5	44.8±2.4	49.2±2.9	35.1±0.6	37.7±0.6
F20	44.4±1.0	47.9±1.1	49.5±1.2	55.3±1.5	42.9±2.6	49.6±2.9	35.0±0.4	37.4±1.0
F40	42.5±2.3	47.5±1.0	48.6±1.8	54.6±2.7	41.9±1.7	48.7±2.5	34.1±0.5	37.2±0.5
BT	42.9±1.7	49.9±0.4	50.3±1.5	55.3±1.5	42.8±2.1	50.0±2.0	36.2±1.5	38.1±1.1

Fresh, fresh leaves; Withered, withered leaves; Rolled, rolled leaves; CTC, CTC leaves ; F20, 20 minutes of fermentation; F40, 40 minutes of fermentation; BT, black tea. All units are in mg g^{-1} (dry weight basis). Values are 'mean±SE' of independent triplicate measurements.
